# Supplementary material for: Prevalence and risk factors of work-related musculoskeletal disorders among shopkeepers in Ethiopia: Evidence from a workplace cross-sectional study
Source: PLoS One. 2024 Mar 21;19(3):e0300934. doi: 10.1371/journal.pone.0300934 (PMC10956884; doi:10.1371/journal.pone.0300934)
Supplement: S1 File — (PDF) [file pone.0300934.s001.pdf]

Date of interview (DD/MM/YYYY:___/_____/________

Questionnaire identification number________________

Kebele_____________________________

**Part 1. Socio-demographic information**			
**Code**	**Questions**	**Response (circle the appropriate option)**	**Skip**
101	What is your age?	____________years.	
102	What is your sex?	1. Female<br>2. Male	
103	What is your religion?	1. Orthodox Christian<br>2. Muslim<br>3. Protestant<br>4. Catholic<br>5. other (specify) _______	
104	What is your current marital status?	1. Married<br>2. Single<br>3. Divorced<br>4. Widowed<br>5. Separated	
105	What is your level of education?	1. Unable to read and write<br>2. Write & read only<br>3. Primary school completed (1-8)<br>4. secondary school complete (9-12)<br>5. Certificate/ diploma / Degree	
106	Monthly salary in Ethiopia Birr?	________________Ethiopian birr (ETB)	
107	Years of your work experience in the current shop?	____________(years)	
108	Number of family size in in house?	_____________person in house	

Part II: Questioners to asses WMSD in all body (9 parts of the human body).				
Code	Have you had any trouble (aches, pains and discomfort) related to your work in the last 12 months? In: (If no, go to the next body region, if yes, please continue)			During the last seven days have you had trouble
201	Neck		1. Yes<br>2. No	1. Yes<br>2. No
202	Shoulder	Right	1. Yes<br>2. No	1. Yes<br>2. No
		Left	1. Yes<br>2. No	1. Yes<br>2. No
203	Upper back		1. Yes<br>2. No	1. Yes<br>2. No
204	Elbows	Right	1. Yes<br>2. No	1. Yes<br>2. No
		Left	1. Yes<br>2. No	1. Yes<br>2. No
205	Lower back		1. Yes<br>2. No	1. Yes<br>2. No
206	Wrists/hands	Right	1. Yes<br>2. No	1. Yes<br>2. No
		Left	1. Yes<br>2. No	1. Yes<br>2. No
207	Hips/thighs	Right	1. Yes<br>2. No	1. Yes<br>2. No
		Left	1. Yes<br>2. No	1. Yes<br>2. No
208	Knees	Right	1. Yes<br>2. No	1. Yes<br>2. No
		Left	1. Yes<br>2. No	1. Yes<br>2. No
209	Ankles/feet	Right	1. Yes<br>2. No	1. Yes<br>2. No
		Left	1. Yes<br>2. No	1. Yes<br>2. No

Part III: Question used to grade musculoskeletal pain status

pain intensity item

1. How would you rate your neck/shoulder/back/back/hip/leg/ankle/wrist pain at the present time? responses 0 to 10. Where 0 is "no pain" and 10 is "pain as bad as it could be"

No pain Pain as bad as it could be

0 1 2 3 4 5 6 7 8 9 10

2. During the past 6 months, how intense was your worst pain? responses 0 to 10. Where 0 is "no pain" and 10 is "pain as bad as it could be"

No pain Pain as bad as it could be

0 1 2 3 4 5 6 7 8 9 10

3. During the past 6 months, on the average, how intense was your pain? responses 0 to 10. Where 0 is "no pain" and 10 is "pain as bad as it could be" (That is your usual pain at times you were experiencing pain.

No pain Pain as bad as it could be

0 1 2 3 4 5 6 7 8 9 10

Disability items

4. About how many days in the past 6 months have you been kept from your usual activities (work school or housework) because of your neck/shoulder/back/back/hip/leg/ankle/wrist pain?

Response: the total number of days disabled = ________________days

5. In the past 6 months how much has the neck/shoulder/back/back/hip/leg/ankle/wrist pain interfered with your daily activities? responses 0 to 10. Where 0 is "no pain" and 10 is "pain as bad as it could be" (That is your usual pain at times you were experiencing pain.

No interference Unable to carry on any activities

0 1 2 3 4 5 6 7 8 9 10

6. In the past 6 months how much has the neck/shoulder/back/back/hip/leg/ankle/wrist pain changed your ability to take part in recreational social and family activities? responses 0 to 10. Where 0 is "no change" and 10 is "extreme change"

No change extreme change

0 1 2 3 4 5 6 7 8 9 10

7. In the past 6 months how much has the neck/shoulder/back/back/hip/leg/ankle/wrist pain changed your ability to work (including housework)? responses 0 to 10. Where 0 is "no change" and 10 is "extreme change"

No change extreme change

0 1 2 3 4 5 6 7 8 9 10

Part IV. Behavioral related characteristics			
Code	Questions	Response code	Skip
301	Your weight in kilogram (kg)	___________kg	
302	Your height in meter (m)	___________m	
303	Do you do any physical exercise?	1. Yes<br>2. No	If No skip to Q NO. 305
304	If **yes** for question number 304, how frequently doing physical exercise?	1. Daily<br>2. Once in a week.<br>3. Twice in a week<br>4. Three times in a week<br>5. Other (_____ in a week)	
	For how much you are doing the exercise?	________________minute	
305	Do you smoke cigarette?	1. Yes<br>2. No	If No skip to Q NO. 306
306	If your answer is **yes** for question, number 305 how many cigarettes do you smoking per day?	___________sticks<br>___________packet	
307	Do you consume any kind of alcohol in the past one year?	1. Yes<br>2. No	If No skip to Q NO. 309
308	If **yes** for question number 307, how frequently you drink alcohol in a week?	1. Daily<br>2. Once in a week.<br>3. Twice in a week<br>4. Three times in a week<br>5. Other (_____ in a week)	
309	Do you experience of chewing khat in the past one year?	1. Yes<br>2. No	If No skip to Q NO. 310
310	If **yes** for question number 309, how frequently chew khat?	1. Daily<br>2. Twice in a week<br>3. Once in a week.<br>4. Other (_____ in a week)	
311	Do you have any medical history of chronic illness?	1. Yes<br>5. No	
312	If **yes** for question 311 specify your medical history of chronic illness	6. ____________________	

Part V: Organizational factors associated with WRMSDs among cashiers			
**Sr. No**	**Questions /variables**	**Coding category**	**Skip to**
401	Where does your shop located?	1. Ground<br>2. First floor<br>3. Second floor<br>4. Third floor<br>**5.** Other___	
402	What type of goods do you sale?)	__________________	
403	For how many working hours per day you have been working in this shop?	________(Hours/day)	
404	For how many working days 'per a week you have been working in this shop?	_________(Day/week)	
405	Do you work in awkward posture (with neck bent 30 degree with support, working with a bent wrist, working with back bent without support, squatting or kneeling) for more than 2 hours?	1. Yes<br>2. No	
406	Do you work sitting/standing in restricted space without changing position?	1. Yes<br>2. No	
407	How many hours do you work on sitting in restricted space?	__________hrs.	
408	How many hours do you work on standing in restricted space?	__________hrs.	
409	Do you have to work in the same motion for less than 30 seconds with no variation every few seconds for two or more hours per day?	1. Yes<br>2. No	
410	Types of setting chair (observe)	1. Fixed<br>2. Adjustable	
411	Do you lifting or carrying heavy loads?	1. Yes<br>2. No	
412	If **yes** for question number 411, approximately how many in Kg?	___________kg	
413	Do you climb stairs or ladders?	1. Yes<br>2. No	

414	If **yes** for question number 413, how frequent you climb stairs or ladders?	_____________	
415	Do you have a habit of taking break (after 1-2 hours of continuous work)?	1. Yes<br>2. No	
416	If the answer for question 415 is 'yes', for how many minutes after work?	_____________ minute	

**Part VI: Psychosocial factors**						
**Questions to measure job stress (Q 501-508)**						
Sr. No	**Questions /variables**	**Job stress score**				
		Never	Rarely	Some times	Often	Very often
501	Conditions at work are unpleasant or sometimes even unsafe.	1	2	3	4	5
502	I feel that my job is negatively affecting my physical or emotional wellbeing	1	2	3	4	5
503	I have high loaded work to do and/or too many unreasonable deadlines.	1	2	3	4	5
504	I find it difficult to express my opinion or feelings about my jobconditions to my superiors.	1	2	3	4	5
505	I feel that job pressures interfere with my family or personal life.	1	2	3	4	5
506	I have adequate control or input over my work duties.	5	4	3	2	1
507	I receive appropriate recognition or rewards for good performance.	5	4	3	2	1
508	I am able to utilize my skills and talents to the fullest extent at work	5	4	3	2	1

Questions to measure job satisfaction (Q 509-518)						
**S. No**	**Questions /variables**	**Job satisfaction score**				
		Very dissatisfied	Dissatisfied	Neutral	Satisfied	Very satisfied
509	I receive recognition for a job well done.	1	2	3	4	5
510	I feel close to the people at work.	1	2	3	4	5
511	I feel good about working at this company.	1	2	3	4	5
512	I feel secure about my job.	1	2	3	4	5
513	I believe management is concerned about me.	1	2	3	4	5
514	On the whole, I believe work is good for myphysical health	1	2	3	4	5
515	My wages are good.	1	2	3	4	5
516	All my talents and skills are used at work.	1	2	3	4	5
517	I get along with my supervisors.	1	2	3	4	5
518	I feel good about my job	1	2	3	4	5

The End

Thank you
