# Supplementary material for: Prevalence and risk factors of work-related musculoskeletal disorders among shopkeepers in Ethiopia: Evidence from a workplace cross-sectional study
Source: PLoS One. 2024 Mar 21;19(3):e0300934. doi: 10.1371/journal.pone.0300934 (PMC10956884; doi:10.1371/journal.pone.0300934)
Supplement: Attachment — Submitted filename: Response to reviewers.docx [file pone.0300934.s003.docx]

**Date:** 15 April 2023

**To**: PLOS ONE, Editorial manager

**Subject**: - Point-by-point response to the editor’s and reviewer’s comments (Manuscript ID**: PONE-D-22-30367**)

**Title**: Prevalence and risk factors of work-related musculoskeletal disorders among shopkeepers in Northwestern Ethiopia: evidence from a workplace cross-sectional study

**Dear Editorial manager,**

We are very grateful for receiving the editor’s and reviewers’ comments on our manuscript, which we believe will improve our manuscript. Following the reviewers' and editors' valuable comments and recommendations, we have revised the work, and we hereby submit the revised work for your consideration along with a point-by-point response to the editor and reviewers.

Kindest regards,

Authors

**A point-by-point response to Editor and Reviewers**

**Thank you all for your constructive and valuable comments and concerns.**

**Note that all changes to the manuscript are included in the track change too.**

**Title**: Prevalence and risk factors of work-related musculoskeletal disorders among shopkeepers in Northwestern Ethiopia: evidence from a workplace cross-sectional study

**Date:** 15 April 2023

**Editor’s comments**

**Mohammad Hayatun Nabi, MBBS, MHSM, MPH, PHD**

*Responses to the editor (****Reference****s correspond to* ***cleaned (revised) version manuscript****)*

| **Comments** | **Responses** | **References** |
| --- | --- | --- |
| 1. Please ensure that your manuscript meets PLOS ONE's style requirements | Thank you for your recommendation. We have accepted the comment and corrected it according to PLOS ONE's style requirements accordingly. | See the cleaned-up manuscript. |
| 2. In your Data Availability statement, you have not specified where the minimal data set underlying the results described in your manuscript can be found. | Dear Editor, Thank you so much for your suggestion. We have included and specified where the minimal data set used in the analysis was found. | See the cleaned-up manuscript. |
| 3. We note that you have indicated that data from this study are available upon request. PLOS only allows data to be available upon request if there are legal or ethical restrictions on sharing data publicly. | Thank you so much for your crucial comments. All authors have accepted the comments and corrected it accordingly. We have uploaded the minimal anonymized data as Supporting Information **S2** as per your comments |  |

**Reviewer: 1**

*Responses to the reviewer:1 (****Reference****s correspond to* ***cleaned (revised) version manuscript****)*

| **Question/comment** | **Responses** | **Reference** |
| --- | --- | --- |
| Thank you for allowing me to review this paper. The article was nicely written and well-described by the authors. However, I have a few questions regarding the manuscript. Kindly find my comments below, | Thank you very much for reviewing our manuscript and providing interesting comments to improve the manuscript. |  |
| Grammatical mistakes evident throughout the manuscript and proper references and writing patterns need to be changed | Thank you so much for your valuable comment. We all authors accepted the comments. In response to your comments and suggestions, we have now revised and improved the language quality and grammatical errors of the manuscript accordingly. The references have now been modified and updated as well. | See the cleaned-up manuscript. |
| **In the abstract section,** |  |  |
| The descriptive portion is usually presented with numbers and percentages for the categorical variables and mean with standard deviation for the continuous variables. I suggest not using a confidence interval to describe the prevalence of a disease. | Thank you for your suggestion. We have now removed the confidence interval (CI) from the description of the prevalence of work-related musculoskeletal disorder. | See the cleaned-up manuscript, line 41 |
| **In the Background section,** |  |  |
| Plenty of studies have been conducted so far regarding WRMDs. Then what will be the novelty of running such a study where we have already aware of the negative impact of a sedentary lifestyle? | The authors are grateful for your comments. Shopkeepers are largely overlooked in research and policy, particularly in developing countries. In Ethiopia, there is a lack of data on the extent of and factors influencing WRMSD among Ethiopian shopkeepers. This data is essential for the design and implementation of prevention and control interventions among shopkeepers. |  |
| **Methodology section** |  |  |
| In the calculation, authors took prevalence as 50%, where studies have been conducted in the same country on different populations or different countries on the same folks. Then why 50%? | Thank you for your comments. The few studies conducted on musculoskeletal disorders among shopkeepers worldwide. To the best of the authors' knowledge (literature review), there is no study conducted on musculoskeletal disorders of shopkeepers in Ethiopia. The authors didn't believe in using the proportion of musculoskeletal disorders of shopkeepers from other countries in calculating the sample size for this study because shopkeepers in Ethiopia work at the cost of their lives. In addition, as many of the shopkeepers in Ethiopia work under informal conditions, workers in this sector are not covered by national labour and other regulations. So, the prevalence of MSDs among shop workers in Ethiopia and elsewhere could be very different. For all the above reasons, all the authors agreed to use 50% in calculating the sample size. |  |
| In total, how many questions were in the questionnaire? | Thank you for your question. We have now attached the questionnaire as supporting information, so the number of questions found in the questionnaire |  |
| What was the average time to complete one interview? | Thank you so very much for rising this point. The average amount of time that it took for an interview to be completed was 40 minutes and we have included the statement in the main manuscript as well. | See the cleaned-up manuscript, lines 244-245 |
| Were all the scales have been validated before use? | All of the instruments have been extensively and repeatedly used in previous studies conducted in in Ethiopia, which were tailored to the local culture and conditions, so we can say that they are validated. |  |
| What were the Cronbach Alpha of the individual scales? | The Cronbach's alpha (α) value for NMQ) was 0.76, and Cronbach's alpha values were 0.75 for pain intensity and 0.73 for disability. Thus, Cronbach's alpha (α) value for the 10-item Job Satisfaction Scale was 0.786, and Cronbach's alpha (α) value for the 8-item Workplace Stress Scale was found to be 0.82. We have now included each Cronbach's alpha values in revised manuscript accordingly. | See the cleaned-up manuscript, lines 258-265 |
| Were the authors included piloting data in the final analysis? | **It was pre-test not pilot**. The pre-test data were not included in the final analysis. They were only used to check the language clarity and to ensure that respondents understood. We have modified the statement in revised manuscript | See the cleaned-up manuscript, line 243 |
| How many data collector was involved in this research? | Three data collectors and one supervisor were involved in our study.  We have now clarified it further in our revised manuscript | See the cleaned-up manuscript, lines 237-238 |
| The authors mentioned that participants completed the questionnaire during the face-to-face interview. But the reason for not taking informed written consent needed to be clarified. This is a serious concern. How could the authors maintain the authenticity of the data and the data collection procedures? It could be a possibility of bias. | Dear reviewer, Thank you so much for your interesting and thoughtful comments. It was a topographical error of original summitted manuscript. The respondents were asked if they were willing to take part in the study, and their written informed consent was obtained. Once they had agreed to participate in the study, the face-to-face interview was conducted to preserve their ability to carry out their normal business activities, as they were busy at work to fill out the administered questionnaires themselves. We all think the comment is absolute, and we have now modified accordingly. Thank you so much once again for your considerate advice. | See the cleaned-up manuscript, lines 283-287 |
| **Result section** |  |  |
| The authors should place age as a continuous variable under the Table 1 section with age groups. | Thank you for your comments. We have now included age as a continuous variable in the Table 1 as per your comment. | See the cleaned-up manuscript, **Table 1** |
| Numbers were mismatched in the "Education Level" variable with the explanation. | Thank you for your appreciated comments. The comment is accepted and corrected accordingly. | See the cleaned-up manuscript, line 299 |
| Three-fourths of the shopkeepers (74.4%) had a low perceived disability of musculoskeletal pain"- should be changed to "Around three-fourths of the shopkeepers (74.4%) had a low perceived disability of musculoskeletal pain". | Thank you for your acceptable recommendation. We have now changed it accordingly | See the cleaned-up manuscript, lines 311-312 |
| What is the justification for using table 4? Why AOR? What were the other variables adjusted here? The authors should exclude that, as it has already been presented in table 6. | Thank you so much for your fruitful comments. We all authors accepted your comment and we have removed table 4 immediately, since as you said already been presented in table 6. Thanks for making redundancy elimination | See the cleaned-up manuscript |
| **Discussion section:** |  |  |
| What is the logic behind evaluating the WRMSDs in the past 12 months? There could be a high possibility of recall biases here. | Dear reviewer, thank you so much for your rising this issue. As we have mentioned in our manuscript this study is the first in its kind in Ethiopia. We have evaluating both 12 months and 7 days WRMSDs. Accordingly, the past 12 months prevalence was 81.1% (n = 507) [95% CI (77.8% to 84.1%)], and the previous 7-days prevalence was 75.2% (n = 470) [95% CI (71.6% to 78.5%)]. In this study, the prevalence of 12 months and 7 days was also almost similar. The problem of WRMSDs was not acute, its chronic. |  |
| In the discussion part, the authors stated, "Besides, measures to prevent injuries and safeguard the health and safety of workers are often lean in many developing nations". Kindly provide some solid references for this statement. | Thank you so much for your comment. The updated with credible references have now been added to the statements as per your comment. | See the cleaned-up manuscript, lines 380-381 |
| **Suggestion:** The study questionnaire could be attached to the manuscript as a supplementary. | Thank you, the comments. The questionnaire has been now attached as a supplementary. All authors gratefully acknowledge all of your comments | See the cleaned-up manuscript |

**Reviewer: 2**

*Responses to the reviewer: 2 (****Reference****s correspond to* ***cleaned (revised) version manuscript****)*

| **Question/comment** | **Responses** | **Reference** |
| --- | --- | --- |
| I have read the "Prevalence and risk factors of work-related musculoskeletal disorders among shopkeepers in Northwestern Ethiopia: evidence from a workplace cross-sectional study" manuscript by Amensisa eta al. The article is well written.  Minor comments | Dear Reviewer, thank you so much for your kind comments. We have given time for your comments and extensively gone through them to revise our manuscript since we found your comments to be very important for the improvements of the manuscript. Now, hopefully, you are interested in our revised manuscript. | See the cleaned-up manuscript |
| **line 10:** corresponding author/ correspondence not "correspondence author" | Thank you for your comment. Following your comment, we have now changed the correspondence word to corresponding accordingly. | See the cleaned-up manuscript, line 11 |
| **Abstract:** |  |  |
| 95% CI of the prevalence does not give sense in the abstract section. Authors may mention this either in the result and preferably in the discussion section as it helps to compare with existing literature. In the abstract, the point prevalence is sufficient. | Dear reviewer, thank you for your insightful comment. All authors have accepted your comment and we have removed the 95% CI of the prevalence reported the abstract section. | See the cleaned-up manuscript, line 41 |
| **Background** is well written. | Thank you very much |  |
| **methods** |  |  |
| under study design and period no need to rewrite the objective as it does not add any value. | Thank you for the acceptable comment. We have removed the rewritten objective from the section accordingly. | See the cleaned-up manuscript |
| in the study setting and area unnecessary details are presented and important information about the total number of shoekeepers is missed. | Thank you for your comment. We have now removed unnecessary details from the section and included the total number of shopkeepers. We have also modified and revised the section as per your comment | See the cleaned-up manuscript, lines 118-123 |
| Line 261 is similar to lines 262 to 263 | Thank you for your insightful comments. We have accepted your comment and removed the repeated sentence accordingly. | See the cleaned-up manuscript, lines 253-254 |
| line 295: What is valid questionnaire? | It was a typo error. We want to mean complete questionnaire. We have now revised and modified the way we explain the sentence | See the cleaned-up manuscript, line 295 |
| Major issues  Lines 138 to 140: Confounding can be addressed by restriction, matching and multivariable analysis. Why do authors prefer restriction as a method of choice and what impacts does this method have on the study's external validity? | Dear reviewer, thank you for raising this important point. We have excluded participants with a history of these conditions as they could potentially bias the results of the study. We have now provided a robust reference to supplement the statement. In our previous manuscript, the use of the word "confounding" was a typographical error, instead we want to say bias. |  |
| Authors claimed that they excluded pregnant women. How did they check pregnancy? | The data in the study was based on self-reporting by the respondents. So, whether the shopkeepers were pregnant or not was reported by the shopkeepers themselves. The data collectors asked them whether they were pregnant or not, and they responded whether they were pregnant or not. |  |
| **Measurement of variables** |  |  |
| 1. what was the pre-test result? | We conducted the pre-test to check the clarity of the language and to ensure that the respondents understood. Not to see the result (prevalence of WRMSD among shopkeepers). |  |
| 2. was NMQ validated in Ethiopia? | The NMQ has been repeatedly used in previous studies conducted in Ethiopian, which were tailored to the local culture and conditions. So, we can say that NMQ was validated. |  |
| 3. Nothing was said about whether the tools used were validated in Ethiopia. | The survey tool has been widely employed to assess musculoskeletal disorders in different settings and is a reliable and accurate method of collecting such evidence. The instrument was also repeatedly used in Ethiopia, which were tailored to the local culture and conditions and we can say equivalent to validated |  |
| 4. line 249 "reduced number of questions"? What were the items deleted? | "reduced number of questions" was typo error. We have now revised and modified the section accordingly. | See the cleaned-up manuscript, *data quality control section* |
| What was the α coefficient of 0.76 for? Authors have used a number of tools and it seems trivial to report a single α coefficient for divergent constructs which measure quite different concepts. | Dear reviewers thank you very much for raising this important issue. We all authors accepted your comment. The previously calculated Cronbach's alpha (α) value of 0.76 was the score for NMQ). As per your crucial comment we have now calculated the Cronbach’s alpha (α) value for each tool. Accordingly, Cronbach's alpha values were 0.75 for pain intensity and 0.73 for disability. Thus, Cronbach's alpha (α) value for the 10-item Job Satisfaction Scale was 0.786, and Cronbach's alpha (α) value for the 8-item Workplace Stress Scale was found to be 0.82. We have included them in our revised manuscript. | See the cleaned-up manuscript, lines 258-265 |
| Line 268, why logistic regression as a method and odds ratio as effect size? As the authors have reported 81.1% prevalence, it would have been good if they use log binomial and report prevalence ratio instead of odds ratio. As it stands now the OR over estimates the PR and the way authors interpreted the odds is not correct. Authors may wish to perform re analysis using log binomial and report prevalence ratio as effect size measure or at least correct the way they interpreted the odds and mention the limitation of using OR over PR for high prevalence report in the limitation section if they wish to stick on logistic regression coming up with convincing reason. | Thank you very much for providing such valuable and thoughtful comments. We have exhaustively reviewed and read different statistical literature. Dear reviewer, your concern was absolute. We all the authors have accepted your comments and performed re analysis using Poisson regression model. Accordingly, we have comprehensively revised, modified, and edited the previously submitted manuscript. Thank you so much for providing such valuable comments for the improvement of our manuscript. All authors gratefully acknowledge your crucial comments. We have learnt a lot from your absolute comment. | See the cleaned-up manuscript, lines 338-358 |
| The authors may wish to annex the ethical clearance paper. | Thank you for the comment. Previously we have summited the ethical clearance paper as “other file”. We have now also included it accordingly. |  |
| The justification for skipping written consent and going to verbal consent is not convincing. | Dear reviewer, Thank you so much for your interesting and thoughtful comments. It was a topographical error of original summitted manuscript. The respondents were asked if they were willing to take part in the study, and their written informed consent was obtained. Once they had agreed to participate in the study, the face-to-face interview was conducted to preserve their ability to carry out their normal business activities, as they were busy at work to fill out the administered questionnaires themselves. We all think the comment is absolute, and we have now modified accordingly. Thank you so much once again for your considerate advice. | See the cleaned-up manuscript, lines 283-287 |
| Physical activity was mentioned as yes/no in the operational definition and reported with frequencies in the result part. how? | Thank you for your question. Physical activity is defined as doing any type of activity for 30 minutes or more at least twice a week. This means that participants who did any type of physical activity ≥ twice a week were marked as 'yes' and others as 'no'. By considering your comment we have made a modification accordingly. | See the cleaned-up manuscript, lines 305-306 |
| The interpretation of odds ratio as relative risk in cross sectional study is trivial. | Thank you for your helpful comments. Your comment has been noted and we have corrected the interpretation immediately. | See the cleaned-up manuscript |
| Authors mentioned that females may have additional strain at home. How did the authors single out whether the MSDS was from exposure to their work or any other source? | Thank you for raising this issue. In Ethiopia, there is a cultural custom that initiates female were lower social class as compared to male. Most of the Ethiopian female are mainly working at home in addition to working outside. Female were only taking care of children, washing clothes and cooking as compared to male in Ethiopia. This may exacerbate or aggravate their musculoskeletal disorders at work. In our study work-related musculoskeletal disorders are pain, ache or discomfort in the musculoskeletal system caused by work or other causes. |  |
| Authors used chronic disease as yes/no? how was it ascertained? | Thank you for your question. Chronic conditions were self-reported conditions such as asthma, diabetes mellitus, stroke, kidney stones and hypertension that can be managed but not cured. We have now included it in the operational definition. | See the cleaned-up manuscript, lines 183-184 |
| line 407 "Accordingly, the likelihood of developing WRMSDs increases with age" To say so, authors were able to use the age score as continuous variable. What was the base of classification for age, work experience and family size? | Thank you very much for the comment. We have accepted your comment and modified the sentence accordingly. We classified the age, work experience, family size and other same variable after reviewing different literature conducted in a similar context. All authors gratefully acknowledge all of your comments, since all of them helped improve our revised manuscript for publication. |  |

**We gratefully acknowledge every one of your insightful and incredibly intriguing remarks.**

**We look forward to your positive response as soon as possible.**

With best regards,

Sincerely yours,

Authors
