# Supplementary material for: Prevalence and risk factors of work-related musculoskeletal disorders among shopkeepers in Ethiopia: Evidence from a workplace cross-sectional study
Source: PLoS One. 2024 Mar 21;19(3):e0300934. doi: 10.1371/journal.pone.0300934 (PMC10956884; doi:10.1371/journal.pone.0300934)
Supplement: Attachment — Submitted filename: PONE-D-22-30367_reviewer_AJF.docx [file pone.0300934.s004.docx]

PLOS ONE

Prevalence and risk factors of work-related musculoskeletal disorders among

shopkeepers in Northwestern Ethiopia: evidence from a workplace cross-sectional study

--Manuscript Draft--

| Manuscript Number: | PONE-D-22-30367 |
| --- | --- |
| Article Type: | Research Article |
| Full Title: | Prevalence and risk factors of work-related musculoskeletal disorders among shopkeepers in Northwestern Ethiopia: evidence from a workplace cross-sectional study |
| Short Title: | work-related musculoskeletal disorders among shopkeepers |
| Corresponding Author: | Amensisa Hailu Tesfaye, MPH  University of Gondar College of Medicine and Health Sciences Gondar, Ethiopia, ETHIOPIA |
| Keywords: | Prevalence, work-related musculoskeletal disorders, shopkeepers, Ethiopia |
| Abstract: | Background: Work-related musculoskeletal disorders (WRMSDs) are the leading cause of disability worldwide. Shopkeepers are prone to developing musculoskeletal disorders as a result of their occupation, but they are largely overlooked in research and policy actions, particularly in developing countries. So far, there is a lack of data on the magnitude and factors influencing work-related musculoskeletal disorders among shopkeepers in Ethiopia. Therefore, the current study aimed to explore the prevalence and risk factors of work-related musculoskeletal disorders among shopkeepers in Northwest Ethiopia.  Methods and Materials: A workplace-based cross-sectional study was conducted from July to August 2022, in Gondar city, Northwest Ethiopia. A multistage sampling technique was used to select 625 shopkeepers. The data were collected using an interviewer-administered standardized Nordic Musculoskeletal Questionnaire. The collected data were entered into Epi Info version 7 and analyzed using Stata version 14. A binary logistic regression was used to analyze the relationship between dependent and independent variables. We set a p-value of 0.05 to ascertain significance of the association and 95% confidence interval (CI) with an adjusted odds ratio (AOR) to evaluate the strength of associations.  Results: The overall response rate was 98.6% (N=625). About half, 321 (51.4%) of the participants were males. The mean (±SD) age of the participants was 36.7 (± 11.5) years. The prevalence of WRMSDs among the shopkeepers in the past 12 months was 81.1% (N = 507) [95% CI:77.8% - 84.1%)]. The most frequently affected body part was lower back (46.6%) followed by the upper back (43.8%), and shoulder (35.4%). Being female [AOR=2.09; 95% CI (1.31-3.33)], being in the age group 30-39 years [AOR=1.92; 95% CI (1.15-3.21)] and >40 years [AOR=3.86; 95% CI (2.06-7.29)], being overweight [AOR=2.04; 95% CI (1.10-3.82)], job stress [AOR=4.04; 95% CI (2.61-6,43)], and prolonged sitting [AOR=1.70; 95% CI (1.06, 2.74)] were the factors associated with work-related musculoskeletal disorders.  Conclusion: This study found a high prevalence of work-related musculoskeletal disorders among shopkeepers. Being female, increasing age, being overweight, job stress, and sitting in the same position for two or more hours have been identified as significant factors for WRMSDs. To curb the onset of WRMSDs, contemporary workplace health and safety programs need to consider the diverse characteristics of the individuals, including gender, age, and BMI. Injury prevention efforts would also benefit from targeting occupational exposures, including static working postures and stress emanating from occupations. |
| Order of Authors: | Amensisa Hailu Tesfaye, MPH |
|  | Gebisa Guyasa Kabito |
|  | Fantu Mamo Aragaw |
|  | Tesfaye Hambisa Mekonnen |

| Additional Information: |  |
| --- | --- |
| Question | Response |
| Financial Disclosure  Enter a financial disclosure statement that describes the sources of funding for the work included in this submission. Review the [submission guidelines](http://journals.plos.org/plosone/s/submission-guidelines#loc-financial-disclosure-statement) for detailed requirements. View published research articles from [PLOS ONE](http://journals.plos.org/plosone/) for specific examples.  This statement is required for submission and will appear in the published article if the submission is accepted. Please make sure it is accurate. | The author(s) received no specific funding for this work. |
| Unfunded studies Enter: The author(s) received no specific funding for this work. Funded studies Enter a statement with the following details: - Initials of the authors who received each award - Grant numbers awarded to each author - The full name of each funder - URL of each funder website - Did the sponsors or funders play any role in the study design, data collection and analysis, decision to publish, or preparation of the manuscript? - NO - Include this sentence at the end of your statement: The funders had no role in study design, data collection and analysis, decision to publish, or preparation of the manuscript. - YES - Specify the role(s) played. |  |
| * typeset |  |
| Competing Interests  Use the instructions below to enter a competing interest statement for this submission. On behalf of all authors, disclose any [competing interests](http://journals.plos.org/plosone/s/competing-interests) that could be perceived to bias this work—acknowledging all financial support and any other relevant financial or nonfinancial competing interests. | The authors have declared that no competing interests exist. |

| This statement is required for submission and will appear in the published article if the submission is accepted. Please make sure it is accurate and that any funding sources listed in your Funding Information later in the submission form are also declared in your Financial Disclosure statement.  View published research articles from [PLOS ONE](http://journals.plos.org/plosone/) for specific examples. |  |
| --- | --- |
| NO authors have competing interests Enter: The authors have declared that no competing interests exist. Authors with competing interests Enter competing interest details beginning with this statement: I have read the journal's policy and the authors of this manuscript have the following competing interests: [insert competing interests here] |  |
| * typeset |  |
| Ethics Statement  Enter an ethics statement for this submission. This statement is required if the study involved:   - Human participants - Human specimens or tissue - Vertebrate animals or cephalopods - Vertebrate embryos or tissues - Field research   Write "N/A" if the submission does not require an ethics statement.  General guidance is provided below. Consult the [submission guidelines](http://journals.plos.org/plosone/s/submission-guidelines#loc-guidelines-for-specific-study-types) for detailed instructions. Make sure that all information entered here is included in the | Ethical clearance was obtained from the Institutional Ethical Review Board (IERB) of the University of Gondar (Reference No: EOHS/IRBN/639//2022). All methods were carried out following the Helsinki Declaration and the guidelines and regulations of the University of Gondar's research ethics review committee. All study participants were informed about the purpose of the study and the importance of their participation in their local language. Thus, the information sheet that clearly shows the research topic, the objectives of the study, confidentiality of the participant's responses, the study benefits, and associated risks was prepared. The information sheet and consent were provided for respondents to read for those who can read and the interviewer read the consent form for those respondents who cannot read. The respondents were then asked if they were willing to take part in the study, and their informed consent was obtained verbally in order to preserve their ability to conduct normal shop operations. They were also allowed to ask any questions they had about the research and to reject or terminate the interview at any time. Each respondent's information was kept private and confidential, and any personal identifiers were removed. Respondents who complained of severe musculoskeletal disorders during the data collection period were advised to look for medical assistance. |

Methods section of the manuscript.

Format for specific study types

Human Subject Research (involving human

participants and/or tissue)

Give the name of the institutional review

board or ethics committee that approved the

study

•

Include the approval number and/or a

statement indicating approval of this

research

•

Indicate the form of consent obtained

(

written/oral) or the reason that consent was

not obtained (e.g. the data were analyzed

anonymously)

•

Animal Research (involving vertebrate

animals, embryos or tissues)

Provide the name of the Institutional Animal

Care and Use Committee (IACUC) or other

relevant ethics board that reviewed the

study protocol, and indicate whether they

approved this research or granted a formal

waiver of ethical approval

•

Include an approval number if one was

obtained

•

If the study involved

non-human primates

,

add

additional details

about animal welfare

and steps taken to ameliorate suffering

•

If anesthesia, euthanasia, or any kind of

animal sacrifice is part of the study, include

briefly which substances and/or methods

were applied

•

Field Research

Include the following details if this study

involves the collection of plant, animal, or

other materials from a natural setting:

Field permit number

•

Name of the institution or relevant body that

granted permission

•

Data Availability

Authors are required to make all data

underlying the findings described fully

available, without restriction, and from the

time of publication. PLOS allows rare

exceptions to address legal and ethical

concerns. See the

[PLOS Data Polic](http://journals.plos.org/plosone/s/data-availability)

[y](http://journals.plos.org/plosone/s/data-availability)

and

[FA](http://journals.plos.org/plosone/s/data-availability#loc-faqs-for-data-policy)

[Q](http://journals.plos.org/plosone/s/data-availability#loc-faqs-for-data-policy)

for detailed information.

No - some restrictions will apply

| A Data Availability Statement describing where the data can be found is required at submission. Your answers to this question constitute the Data Availability Statement and will be published in the article, if accepted. |  |
| --- | --- |
| Important: Stating ‘data available on request from the author’ is not sufficient. If your data are only available upon request, select ‘No’ for the first question and explain your exceptional situation in the text box. |  |
| Do the authors confirm that all data underlying the findings described in their manuscript are fully available without restriction? |  |
| Describe where the data may be found in full sentences. If you are copying our sample text, replace any instances of XXX with the appropriate details.   - If the data are held or will be held in a public repository, include URLs, accession numbers or DOIs. If this information will only be available after acceptance, indicate this by ticking the box below. For example: All XXX files   are available from the XXX database (accession number(s) XXX, XXX.).   - If the data are all contained within the manuscript and/or Supporting Information files, enter the following: All relevant data are within the manuscript and its Supporting Information files. - If neither of these applies but you are able to provide details of access elsewhere, with or without limitations, please do so. For example:   Data cannot be shared publicly because of [XXX]. Data are available from the XXX Institutional Data Access / Ethics Committee (contact via XXX) for researchers who meet the criteria for access to confidential data.  The data underlying the results | Authors presented the data in the main paper and made available from the corresponding author on a reasonable request via e-mail address: amensisahailu@gmail.com. |

| presented in the study are available from (include the name of the third party and contact information or URL).  • This text is appropriate if the data are owned by a third party and authors do not have permission to share the data.  * typeset |  |
| --- | --- |
| Additional data availability information: |  |

Manuscript	[Click here to access/download;Manuscript;Shopkeepers plose](https://www.editorialmanager.com/pone/download.aspx?id=32195006&guid=4a4834da-aae5-491f-b459-1346c3dbec4b&scheme=1)

[one1.docx](https://www.editorialmanager.com/pone/download.aspx?id=32195006&guid=4a4834da-aae5-491f-b459-1346c3dbec4b&scheme=1)

1 **Prevalence and risk factors of work-related musculoskeletal disorders among**
2 **shopkeepers in Northwestern Ethiopia: evidence from a workplace cross-sectional study** 3	Amensisa Hailu Tesfaye^1^*, Gebisa Guyasa Kabito^1^, Fantu Mamo Aragaw^2^, and Tesfaye Hambisa 4	Mekonnen^1^
5 **Authors’ affiliations:**
6 ^1^Department of Environmental and Occupational Health and Safety, Institute of Public Health, 7 College of Medicine and Health Sciences, University of Gondar, P.O. Box 196, Gondar, Ethiopia
8 ^2^Department of Epidemiology and Biostatistics, Institute of Public Health, College of Medicine
9 and Health Sciences, University of Gondar, P.O. Box 196, Gondar, Ethiopia
10* Correspondence author
11 **Email address of authors:**
12 Amensisa Hailu Tesfaye: amensisahailu@gmail.com
13 Gebisa Guyasa Kabito: gebeguyasa4@gmail.com
14 Fantu Mamo Aragaw: fantuma3@gmail.com
15 Tesfaye Hambisa Mekonnen: tajan2022@gmail.com

16

17

18

19

20

# Abstract

**Background:** Work-related musculoskeletal disorders (WRMSDs) are the leading cause of disability worldwide. Shopkeepers are prone to developing musculoskeletal disorders as a result of their occupation, but they are largely overlooked in research and policy actions, particularly in developing countries. So far, there is a lack of data on the magnitude and factors influencing work-related musculoskeletal disorders among shopkeepers in Ethiopia. Therefore, the current study aimed to explore the prevalence and risk factors of work-related musculoskeletal disorders among shopkeepers in Northwest Ethiopia.

**Methods and Materials:** A workplace-based cross-sectional study was conducted from July to August 2022, in Gondar city, Northwest Ethiopia. A multistage sampling technique was used to select 625 shopkeepers. The data were collected using an interviewer-administered standardized Nordic Musculoskeletal Questionnaire. The collected data were entered into Epi Info version 7 and analyzed using Stata version 14. A binary logistic regression was used to analyze the relationship between dependent and independent variables. We set a p-value of <0.05 to ascertain significance of the association and 95% confidence interval (CI) with an adjusted odds ratio (AOR) to evaluate the strength of associations.

**Results:** The overall response rate was 98.6% (N=625). About half, 321 (51.4%) of the respondents were male. The mean (±SD) age of the respondents were 36.7 (± 11.5) years. The prevalence of WRMSDs among the shopkeepers in the past 12 months was 81.1% (N = 507). The most frequently affected body part was lower back (46.6%) followed by the upper back (43.8%), and shoulder (35.4%). Being female (p=0.002), being in the age group 30-39 years and ≥40 years , being overweight, job stress, and prolonged sitting were the factors associated with work-related musculoskeletal disorders.

**Conclusion:** This study found a high prevalence of work-related musculoskeletal disorders among shopkeepers. Being female, increasing age, being overweight, job stress, and sitting in the same position for two or more hours have been identified as significant factors for WRMSDs. To curb the onset of WRMSDs, contemporary workplace health and safety programs need to consider the diverse characteristics of the individuals, including gender, age, and BMI. Injury prevention efforts would also benefit from targeting occupational exposures, including static working postures and stress emanating from occupations.

**Keywords:** Prevalence, work-related musculoskeletal disorders, shopkeepers, Ethiopia

# Background

Work-related musculoskeletal disorders (WRMSDs) are defined as a group of painful disorders of the locomotor system that affect the muscles, nerves, tendons, joints, cartilage, ligaments, spinal discs, bones, and related soft tissues in all parts of the body and have been empirically linked to workplace risk factors [1-3]. In developing nations, the rate of WRMSDs is quite high due to poor working conditions and a lack of an effective work-related injury prevention and control programs [4]. WRMSDs can progress from mild to severe disorders over time and are seldom life-threatening, but they reduce the quality of life for a large proportion of the world's working population [1].

Musculoskeletal disorders (MSDs) caused by occupational exposure have become a major health problem around the world. [5]. Globally, WRMSDs are a leading contributor to work-related injury and disability [6, 7]. They have become a global public health concern [8, 9] due to their overall consequences, which include decreased productivity [10, 11], work absenteeism [12], limited mobility, resulting in early retirement from work, lower levels of well-being, physical disability, and mental disorders [1, 7, 13, 14]. A recent analysis of Global Burden of Disease (GBD) 2019 data showed that about 1.71 billion people globally live with MSDs and these disorders are the biggest contributors to years lived with disability (YLDs) worldwide, with approximately 149 million YLDs, accounting for 17% of all YLDs worldwide [15]. The burden of MSD is increasing across the entire Sub-Saharan population [16]. WRMSDs and other musculoskeletal conditions are less prioritized and empirically underrepresented in low and middle-income countries (LMICs), particularly in Ethiopia, due to a focus on more pressing and life-threatening health issues such as poverty and infectious diseases [17].

According to the latest estimates from the Labour Force Survey (LFS), 470,000 workers were affected by WRMSDs in 2020/21. This represents a prevalence rate of 1,420 per 100,000 workers and accounts for 28% of all work-related ill health [1]. As per reports from Canada and the Netherlands [18, 19], the prevalence of MSDs ranges from 29% to 74.5%. A high prevalence of WRMDs has been recorded among workers who are exposed to manual labor, work in unusual and restricted postures, repetitive and static work, vibrations, and poor psychological and social conditions [5, 20]. Shopkeepers work in physically demanding and perilous working conditions, which lead to the development of WRMSDs [21]. Studies from Lahore, Pakistan [22] indicated that 56.25% of shopkeepers suffered from low back pain, with 30% experiencing severe pain and 58% having difficulty at work. According to a study conducted in India [21] 70.60% of shopkeepers reported musculoskeletal pain, with the highest prevalence found in the low back area (57.33%), followed by the knee (30%). WRMSDs are a problem in all African countries, with the prevalence of the musculoskeletal disease ranging from 15% to 93.6% [23]. In Ethiopia, the prevalence of WRMSDs ranges from 35% to 74.5% according to few studies conducted in the working population [24]. However, studies on WRMSDs among shopkeepers are lacking.

Shopkeepers are people who run a shop as their sole source of income [21]. They frequently work long and erratic hours, including at night and on weekends [25]. Thus, due to the nature of the shopping industry, shopkeepers are regularly vulnerable to risk factors such as prolonged standing, frequent lifting, awkward posture, and constant carrying of stock that are associated with the involvement of WRMSDs [21, 22]. An extended sitting position is also common when customers are not visiting shopping centers, potentially increasing their risk of developing musculoskeletal conditions [21]. The occurrences of WRMSDs also influenced by personal or individual factors [5, 26]. Depending on the previous investigations, these factors include age, gender, alcohol/tobacco consumption, physical activity, endurance, anthropometry, and chronic illness, as well as educational level [5, 26-30]. Furthermore, psychosocial risk factors related to workplace emotional perception influence the risk of developing WRMSDs [4, 26, 31].

Evidence supporting labor rights and promoting safe working conditions is scarce at a global level. Specifically, shopkeepers are continuing to work in hazardous conditions that jeopardize their health, safety, and well-being. In particular, they are at a high risk of developing WRMSDs, but they are largely overlooked in research and policy actions, particularly in developing countries. In Ethiopia, minimal attention has been paid to implementing health and safety programs in the informal economies, particularly in business sectors such as the shopping industries. So far, there is a lack of data on the magnitude and factors influencing WRMSDs among shopkeepers in Ethiopia. Therefore, the current study aimed to determine the prevalence and associated factors of WRMSDs among shopkeepers in Northwest Ethiopia. The findings of this study contribute to the achievement of the United Nations 2030 Agenda of Sustainable Development Goals (SDGs), specifically SDG 8.8 (protect labor rights and promote safe and secure working environments for all workers). It also provides relevant data for policymakers and concerned stakeholder to design and implement prevention and control measures help alleviate further episodes.

**Methods and Materials**

# Study design and period

A workplace-based cross-sectional study was conducted from July to August 2022 to assess prevalence and risk factors of work-related musculoskeletal disorders among shopkeepers in Northwestern Ethiopia.

**Study setting**

The study was carried out among shopkeepers in Gondar city. Gondar City is located 727 km away from Addis Ababa, the capital of Ethiopia. The city is situated at a latitude and longitude of 12°36’N and 37°28’E with an elevation of 2133 m above sea level [32]. According to the 2013 Ethiopian Central Statistics Agency (CSA) report, the population of Gondar city was estimated to be 323,875 by 2015 and to have increased to 360,600 in 2017 [33]. The majority of the city’s economic activities are centered on trading, and many city residents are involved in the trading sector to generate their income. Shopping accounted for a larger portion of the city's total business [34]. Administratively, the city is divided into six sub-cities: Fasil, Jantekel, Arada, Zobel, Maraki, and Azezo. There were 10,432 shops in all sub-cities during the data collection period.

# Source and study populations

All shopkeepers in Gondar city were the source population, whereas the randomly selected shopkeepers working in selected sub-cities were the study population. Shopkeepers who had worked for at least 12 months prior to the study were eligible for this study, while those who had a history of accidents involving the musculoskeletal system (fractures of the spine or limbs due to a car accident or fall), spinal surgeries and major surgeries on any part of the body, congenital anomalies such as spinal and limb anomalies, and pregnant women were excluded.

# Sample size determination and sampling procedure

The sample size was calculated using a single population proportion formula [35]: with the following assumptions: 5% margin of error (d), proportion (p) of work-related musculoskeletal disorders among shopkeepers 50% (no previous study in a similar context), and Zα/2 =the value of the standard normal curve score corresponding to the given confidence interval. Accordingly, based on a single population proportion formula: -

Where: n = initial sample size, Zα/2 = 1.96 corresponding to 95% confidence level, p = proportion = 50%, d = Margin of error = 5% = 0.05

Since a multi-stage sampling technique was used in this study, the calculated sample size was multiplied by a design effect of 1.5 to account for the estimated sampling variance. Using a design effect of 1.5 to 2.0 is authorized in the absence of previous studies in a similar context [36]. Consequently, the overall sample size for this study was 634 after adding a 10% contingency for the non-response rate.

A multistage sampling technique was used to select study participants. All the sub cities were first considered (included in chance) for a random selection, but only two (Maraki & Arada) sub cities were chosen at random using the lottery method due to feasibility concerns. Shops for each selected sub cities were allocated by using proportional allocation; by assuming that in each shop there was one shopkeeper. During the study period, there were 3459 shops in the Arada sub-city, and 2559 shops in the Maraki sub-city. Therefore, the required sample size of shopkeepers from the selected sub cities were 364 from Arada sub city and 270 from Maraki sub city. A systematic random sampling technique was used to select shops in selected sub-cities. The first shops were chosen through a lottery method. When two or more shopkeepers were found in a shop, one of them was chosen by lottery methods (**Figure 1)**.

# Operational definition

**Work-related musculoskeletal disorders (WRMSDs):** a self-reported pain, ache, or discomfort in any part of the neck, shoulder, upper back, lower back, hip/thigh, knee/leg, ankle/foot, or wrist/hand at any time in the previous 12 months [37].

**High perceived severity:** a pain intensity score of 50 or greater and less than 3 disability points [38]

**Low perceived severity:** a pain intensity score of less than 50 and less than 3 disability points [38].

**High perceived disability:** a pain disability point score of 3–6 points [38].

**Low perceived disability:** a pain disability point score of less than 3 points [38].

**Perceived job stress:** a score of at least 21 on the workplace-stress scale [39].

**Perceived job satisfaction:** the total score of at least 32 on the generic job satisfaction scale [40].

**Body mass index:** weight in kilograms divided by square of height in meters (kg/m2) categorized as underweight when body mass index (BMI) <18.50, normal (healthy) when BMI = 18.50–24.99, overweight/obese when BMI ≥25.00 [4].

**Doing physical exercise:** exercising or doing any kind of sports activity at least two times per week with a duration of at least 30 minutes [4, 41].

**Cigarette smoker:** It is practice of smoking cigarette by shopkeepers for at list one sticks of cigarette per day [42].

**Alcohol drinker:** the consumption of any kind of alcohol at least two times per week [42].

**Khat chewer:** chewing khat three times a week for at least 12 months [43, 44].

**Awkward postures:** working for two or more hours per day with the neck bent without support; working with a bent wrist; working with the back bent without support; squatting and kneeling [5].

**Adjustable sitting chairs:** chairs have wheels or castors suitable for the floor surface, have adjustable seat height and available within a distance of at least 1.5 meter [45].

**Taking rest breaks:** take brief rest breaks every 60 to 120 minutes. During these breaks, stand up, move around, and do something else, or get a soft drink or a cup of coffee or tea [4].

**Repetitive work:** repeated the same motion for less than 30 seconds or no variation every few seconds for two or more hours per day [46].

**Static posture:** sitting or standing in a restricted space for two or more hours without changing positions [46].

## Data collection tool and procedure

The data were collected using a standardized Nordic Musculoskeletal Questionnaire (NMQ) [37] after the investigators reviewed and modified various related literature based on the study objectives [4, 5, 20, 21, 38, 47]. The questionnaire was first prepared in English and then translated into the local Amharic language and vice versa to ensure consistency. Then a face-to-face interview was conducted by using pre-tested and structured questionnaire at the respondents’ workplace. The questionnaire was divided into four separate domains. The first domain was designed to collect socio-demographic data such as age, gender, educational status, marital status, religion, years of current work experience, monthly income, and family size of respondents. The second domain of the questionnaire is focused on gathering information on musculoskeletal disorders at work. The NMQ has been used to measure the magnitude of pain, ache, or discomfort in the neck, shoulder, upper back, lower back, hip/thigh, knee/leg, ankle/foot, wrist/hand, and elbow musculoskeletal disorders [37]. The NMQ has been validated and proven to be reliable in a variety of languages with acceptable psychometric properties [48, 49]. In Ethiopia, previous studies have made extensive use of the NMQ to investigate the public health problems associated with the disorders in different populations [5, 28, 50]. The questionnaire's third domain contains information used to assess behavioral and psychosocial factors such as cigarette, BMI (kg/m2), physical activity (yes/no), smoking (yes/no), alcohol consumption (yes/no), chewing khat (yes/no), history of chronic illness (yes/no), perceived severity and disability of musculoskeletal pains, as well as perceived job satisfaction and perceived job stress.

The 7-items Von korff et al. [38] questionnaire was used to assess the shopkeepers’ perceived severity and disability of musculoskeletal pain. This instrument is graded on a scale of 0 to 10 and has a total score of 100. The perceived severity of pain was calculated by the formula = (((response question 1) + (response question 2) + (response question 3)) / 3) * 10 and perceived disability score = (((response question 5) + (response question 6) + (response question 7)) / 3) * 10 and disability points = (points for disability days or question number 4) + (points for disability score). A final score was categorized into two with a score of <50 or disability points 3 = 0 (low perceived severity), a score of ≥50 or disability points < 3 = 1 (high perceived severity) and perceived disability = disability points < 3 = 0 (low perceived disability) and disability points ≥ 3 = 1 (high perceived disability). To assess shopkeeper job satisfaction, a 10-item generic job satisfaction scale questionnaire was used [40]. The scale comprised ten questions ranging from 1 to 5 each item and ranged from very dissatisfied, dissatisfied, neutral, satisfied and very satisfied, according to their occurrence respectively. The scale had 10 items with a rating of 1 to 5, and the responses ranged from very dissatisfied, dissatisfied, neutral, satisfied and very satisfied, depending on how frequently they occurred in the month before to the survey and then summing up all 10 items. The scale produced a single ranking, with high scores indicated higher job satisfaction vice versa. Perceived job-related stress of the participants was collected using the 8-item workplace stress scale questionnaire [39]. The scale comprised eight questions ranging from 1 to 5 each item and ranged from never, rarely, sometimes, often and very often, according to their occurrence respectively. The 8- item workplace stress scores are obtained by reversing scores on three positive items, e.g. 5 = 1, 4 = 2, 3 = 3, etc., and then summing up all 8 items. Items 6, 7 and 8 are positive items. The scale produced a single ranking, with high scores indicated higher stress levels and vice versa. All instruments used in the current study have been employed in previous studies conducted in the country’s context [4, 5, 29]. The final domain of the tool incorporates work-related environment and ergonomics characteristics of study participants, including location of shop, type of shop, working hours (per day and peer week), working postures (sitting, standing, awkward posture, and repetitive work), the practice of taking breaks, and type of sitting chair.

## Data quality assurance

To ensure the quality of the data, the questionnaire was first developed in English and then translated into Amharic and back to English by language experts and physiotherapist. Three data collectors from graduating classes of environmental and occupational health and safety students, as well as an MPH environmental health supervisor who worked in Gondar city, participated in the data collection process. One day training was given for the data collectors regarding the data collection tool, techniques of interview, research ethics, and selection methods of study participants. We also conducted a pretest one week before the actual data collection period on 5% of the sample size (on 32 shopkeepers) in the Debark, nearby Gondar city, to check the response, language clarity, appropriateness, and consistency of the instrument. Based on the findings of the pretest analysis, we modified some words and misinterpretations; reduced the number of questions without affecting what was indented to be measured; corrected some other ambiguities; and clarified some unclear and ambiguous questions. Problems encountered during the data collection process were resolved through on-the-spot discussions with the principal investigator, supervisor, and data collectors. Besides, the principal invigorator supervised the interview process. The data collectors checked the collected data immediately after finishing the questionnaire before leaving the shop. The investigators double-checked the completeness and accuracy of all completed questionnaires daily. Finally, nine incomplete and invalid data were removed from the study.

## Data management and statistical analyses

Each questionnaire was checked for completeness and consistency on a daily basis. The collected data were manually entered into Epi Info version 7 for cleaning, editing, coding, and checking for missing values and exported into Stata version 14 for further analysis.

Descriptive statistics were computed and presented using frequencies, percentages, means, standard deviations, tables, and graphs. Multicollinearity among independent variables was assessed using variance inflation factor (VIF) and found to be acceptable (all variables had VIF

<5 values) (Ref). The reliability of the questionnaire was tested using Cronbach's α , and the value of the α coefficient was found to be 0.76. A binary and multivariable logistic regression models were fitted. In the binary logistic regression, variables with p-value < 0.2 were considered for the multivariable logistic regression model using the enter variable selection method. The Hosmer-Lemeshow (HL) test was used to check model fitness, and we found the model was a good fit (p-value=0.5491) [51]. Significance was set at p<0.05 and adjusted odds ratio (AOR) with a 95% confidence interval (CI), model fitness indices was also computed using the Hosmer-Lemeshow (HL) test.

## Ethical approval and consent to participate

Ethical clearance was obtained from the Institutional Ethical Review Board (IERB) of the University of Gondar (**Reference No. EOHS/IRBN/639/2022**). The study followed the ethical principles outlined by the Helsinki Declaration and the guidelines (Ref). All study participants were informed about the purpose of the study and the importance of participation in the study. Participation in the study was voluntary. The right of the respondent to withdraw from the study at any time was disclosed clearly. Each respondent's information was kept private and confidential. . Respondents who complained of severe musculoskeletal disorders during the data collection period were advised to look for medical assistance.

## Results

## Socio-demographic characteristics of study participants

A total of 625 questionnaires were analyzed, yielding a response rate of 98.6%. About half, 321 (51.4%) of the respondents were male. The mean (±SD) age of the respondents was 36.7 (± 11.5) years. The majority, 375 (60.0%) of them were married, In terms of educational level, 13 (2.1%) of them couldn't read or write, while 250 (40.0%) had an elementary education, 187 (45.7%) had a secondary school education, and the remaining 97 (15.5%) had an above-secondary school certificate. Two hundred twenty-three (35.7%) of respondents had worked in the shop for 6 to 10 years, and 98 (15.7%) of them had worked for more than 10 years **(Table 1)**.

## Behavioral and psychosocial characteristics of study participants

According to WHO classification, the majority of shopkeepers, 443 (70.9%), had a normal BMI (18.5-24.9 kg/m2), while 151 (24.2%) had an overweight BMI (> 18.5 kg/m2). Only 73 (11.7%) of shopkeepers engage in physical exercise twice or more per week. Fifty-six (9%) of them have cigarette smoking behaviors, and 181 (29%) of them consume alcohol at least twice a week. Eighty (12.8%) of shopkeepers have a medical history of chronic illnesses such as asthma (n = 26), hypertension (n = 15), diabetes mellitus (n = 33), heart disease (n = 2), and kidney stones (n = 4). The majority, 498 (79.7%) of the shopkeepers, had a low perceived severity of musculoskeletal pain, while 127 (20.7%) had a high perceived severity of it. Three-fourths of the shopkeepers (74.4%) had a low perceived disability of musculoskeletal pain, whereas about one-fourth of them

(25.6%) had a high perceived severity of the pain. In terms of psychosocial characteristics, 173 (27.7%) of the shopkeepers were dissatisfied with their current job, and 401 (64.2%) of them described being perceived to be stressed as a result of their job (**Table 2**).

## Work-related environment and ergonomic characteristics of study participants

Small local shops (wholesalers) are the most common type of shop in the study (30.7%), and the majority of shopkeepers (84.5%) work on the ground floor. The majority, 447 (71.5%) of the participants worked more than 8 hours per day at their job, and more than half, 346 (55.4%) of them worked more than five days per week. More than two-thirds (61.9%) of shopkeepers work for two or more hours per day with a bent neck without support, a bent wrist, a bent back without support, squatting and kneeling. Four hundred and thirty-nine (70.2%) of the shopkeepers explained that they had not taken any breaks at their workplace. Only 7.5% of those who responded to the survey had used adjustable sitting chairs. The majority of shopkeepers, 62.6% and 58.4%, respectively, sat and stood in a confined space for two or more hours per day without changing position. The majority of respondents, 393 (62.9%), stated that their job required repetitive movements (**Table 3**).

## Prevalence of work-related musculoskeletal disorders among shopkeepers

This study revealed that the overall prevalence of self-reported one or more work-related musculoskeletal disorders among shopkeepers in northern Ethiopia during the previous 12 months was 81.1% (n = 507) [95% CI (77.8% to 84.1%)], and the previous 7-days prevalence was 75.2% (n = 470) [95% CI (71.6% to 78.5%)]. Lower back pain (46.6%), upper back pain (43.8%), and shoulder pain (35.4%) were the most frequently reported complaints among work-related musculoskeletal disorders in the previous 12 months (**Figures 2 and 3**). There was a significant difference in the 12 months cumulative prevalence of work-related musculoskeletal disorders among males (77.9%) and females (84.5%) shopkeepers. **Table 4** shows the result of the chisquare test. The p-value obtained was <0.05 (χ2= 4.5189), showing a significant association between musculoskeletal disorders and sex differences. The calculated odds ratio (OR) (OR = 2.07, 95% CI= 1.30-3.30) showed that female shopkeepers were 2.07 times more likely to have musculoskeletal disorders compared with male shopkeepers. Furthermore, among work-related musculoskeletal disorders, lower back pain and upper back pain showed a significant difference in prevalence between males and females (**Table 5**).

## Factors associated with work-related musculoskeletal disorders

The association between independent variables and the outcome variable (work-related musculoskeletal disorders) was examined using a binary logistic regression model, and variables that were significant at a p-value of <0.2 in the bivariable logistic regression analysis were entered into a multivariable logistic regression analysis to control for confounding variables with the outcome variable at a p-value of <0.05. After controlling for confounding variables in

In the multivariable logistic regression analysis sex, age, body mass index (BMI), perceived job stress, and prolonged sitting in a confined space for two or more hours per day without changing position were significant factors determining the manifestations of WRMSDs among shopkeepers in northwestern Ethiopia.

Female shopkeepers had a two-fold increase in the likelihood of developing work-related musculoskeletal disorders when compared to their male counterparts [AOR=2.09; 95% CI (1.313.33)]. Age of the respondents was associated with work-related musculoskeletal disorders. The likelihood of suffering from musculoskeletal disorders was increased by two-fold among shopkeepers with age range from 30 to 39 years as compared to those with an age range of 20 to 29 years [AOR=1.92; 95% CI (1.15-3.21)], while shopkeepers ≥40 years of age were four times more likely to develop musculoskeletal disorders than those whose age ranged from 20 to 29 years [AOR=3.86; 95% CI (2.06-7.29)]. The occurrence of work-related musculoskeletal disorders was also significantly influenced by participant body mass. Shopkeepers with an overweight body mass index (≥25 kg/m^2^) were 2.04 times more likely to develop musculoskeletal disorders than shopkeepers with a normal body mass index (18.5-24.9 kg/m^2^) [AOR=2.04; 95% CI (1.10-3.82)]. Moreover, the chances of suffering from musculoskeletal disorders among shopkeepers who had perceived job stress were 4.09 times higher as compared with those who had no job stress [AOR=4.04; 95% CI (2.61-6,43)]. Shopkeepers who reported long periods of seating in their workplace were 1.70 times more likely to have musculoskeletal disorders than those who did not report long periods of seating [AOR=1.70; 95% CI (1.06, 2.74)] **(Table 6)**.

**Discussion**

The prevalence of work-related musculoskeletal disorders among the shopkeepers during the previous 12 months was 81.1%. The prevalence during the past 7 days has been reported in 75.2% of the study participants. This finding highlights that exposure to workplace factors that are associated with the experience of work-related musculoskeletal conditions among shopkeepers is prominent. This implies that in Ethiopia, minimal attention has been given to implementing health and safety programs in the informal economies, notably in the business sectors, such as the shopping industries.

The current study showed that the low back is the most frequently affected body part (46.6%). The reports in India (57.33%) [21] and Pakistan (56.25%) [22], however, documented a higher prevalence compared to our finding. The data from Pakistan explicitly focused on low back pain, whereas our survey assessed the overall prevalence as the primary objective, and the prevalence from India was assessed over the 3 months duration, which could explain the contradictory findings. Whereas 43.8% and 35.4% of the shopkeepers in our survey indicated they suffered from upper back and shoulder pain, respectively, which is higher than the results from the study in India (10% versus 15%). Such heterogeneity is most likely caused by differences in sample size.

Our survey showed a knee pain prevalence of 32%, which is comparable to the prevalence rate of 30% in India [21]. The nature of the activities associated with shop keeping, such as prolonged sitting and standing in a static position, which may result in the development of muscle and skeletal system dysfunction is almost similar everywhere. Besides, measures to prevent injuries and to safeguard the health and safety of workers are often lean in many developing nations.

In the multivariable analysis, we found a significant association between sex and the experience of WRMSDs among shopkeepers. Hence, females had a greater chance of developing WRMSDs. This result is supported by previous studies in Ethiopia [52], Ghana [53], India [54], and China [55]. This could be due to physiological, morphological, or sociocultural differences between males and females. Females have less muscle strength and a higher percentage of type 1 muscle fibers (weaker in nature) than males of the same size, making them more prone to MSDs [56, 57]. Moreover, most work environments (e.g., surface height, tool design, equipment size) are usually designed to fit anthropometric dimensions and the strength of a male’s capabilities, while females are also engaged in the same work setup, which may place additional strain on female bodies.

Furthermore, females’ home-work interface, such as handling various household-related tasks (cleaning, cooking, child care, laundry, etc.), may put additional physical and psychological burdens on them; aggravating the probability of having WRMSDs than males [58].

Our analysis outlines that age is another significant factor for WRMSDs in this study. This finding corroborates other studies [5, 59, 60]. Accordingly, the likelihood of developing WRMSDs increases with age. This could be due to the fact that with increasing age, musculoskeletal tissues would be vulnerable to bone fragility, loss of cartilage resilience, reduced ligament elasticity, loss of muscular strength, and fat redistribution, decreasing the ability of the tissues to carry out their normal functions [61]. Scientific evidence also suggests that aging-related biological changes, such as degenerative changes in muscles, tendons, ligaments, and joints, may contribute to the pathogenesis of MSDs [62]. As a result, increasing age may increase the susceptibility of musculoskeletal tissues to accumulated workload overtime that increases the probability of experiencing WRMSDs [63].

In our study analysis, there has been found a significant relationship between WRMSDs and BMI. Overweight shopkeepers were 2.04 times more likely to develop WRMSDs than those with a normal BMI. Similar findings were found in studies conducted in India [64, 65] and Indonesia [66]. The plausible reason might be that being overweight is one of the health risk factors with the potential to aggravate the occurrence of WRMSD through increasing physiological and mechanical load on tissues [65]. A high amount of adipose tissue around the muscles and joints due to increased BMI can also limit a person’s movements, thereby stressing musculoskeletal tissues and potentially resulting in musculoskeletal pain [65, 67]. Thus, being overweight causes fatigue and postural discord, which could lead to severe MSDs [68].

Our finding indicated that shopkeepers who experienced job-related stress were four times more likely to suffer from musculoskeletal disorders than those who did not. This finding is in line with the results reported in Ethiopia [52, 69] , Iran [70], and India [71]. The plausible reason could be due to the fact that job stress causes an increase in the release of catecholamine and cortisol hormones, which negatively impacts the structure and function of muscles, tendons, and ligaments [72]. Scientific evidence quantifies that high cortisol concentrations in the blood have an effect on protein and carbohydrate metabolism in muscle tissue. Increased cortisol levels cause muscle weakness by increasing the release of gluconeogenesis substrates from peripheral tissues. As a result, job stress can result in increased muscle tension and blood flow restrictions, leading to musculoskeletal disorders and chronic pain [73, 74]. In addition, individuals' behaviors might be changed under stress, resulting in a faster rate at which they complete tasks. This may lead to faster and more forceful responses during work tasks, with an increased chance of exposure to biomechanical hazards that may aggravate MSDs [75].

Prolonged static sitting is a growing occupational health risk in the workplace, and it is a risk factor for a variety of health problems [76]. Our analysis found that shopkeepers who were seated for long periods of time were more prone to work-related musculoskeletal complaints. Similar results were reported in other studies [22, 76]. This could be due to the fact that prolonged sitting can put strain on muscles, tendons, and ligaments, as well as raise disc pressure. This increases the risk of pain, discomfort, and injuries associated with postural stress disorders, joint compression, and softtissue (muscles, tendons, and ligaments) injuries [77, 78]. In addition, sitting for a long period of time prime to monotonously low overall energy consumption. This may lead to a situation where the body’s energy demand for the back region is well below what is recommended for a healthy lifestyle. In this way the combination of a low metabolic level and lowered blood circulation can eventually lead to muscle degeneration and osteoporosis. As well, prolonged sitting reduces the amount of oxygen that reaches muscles and organs [76]. Therefore, extended periods of sitting have a detrimental effect on the emergence of MSDs.

## Strengths and limitations of the study

This pioneering study examines the prevalence and risk factors of WRMSDs among Ethiopian shopkeepers. The study contributes evidence from a relatively large sample with very good response rates (98.6%). This study offers significant insights into musculoskeletal conditions and the factors affecting them in the context of retailer. The findings of this study also provide key evidence for stakeholders and policymakers that they can use to develop and put into practice prevention and control strategies that support a safe working environment for formal and informal sectors. It also encourages other researchers to conduct additional research into the relationships between a variety of workplace factors and the development of various types of pain in work-related musculoskeletal systems. However, there are limitations to which caution is due when interpreting the findings. First, due to the cross-sectional study design, establishing causal links between variables and WRMDs was impossible. Second, since the study employed a self-report assessment method (questionnaire survey), reporting bias and recall bias could influence the results. we didn't conduct a posture analysis to determine how much each participant had been exposed to workplace ergonomic factors due to concerns about the feasibility of the large sample size used in the study; instead, we looked at potential risk factors that could lead to WRMDs. We recommend that future research validate the variables influencing the occurrence of musculoskeletal disorders by employing a robust study design, such as prospective or longitudinal case control. Future studies also recommend performing posture analysis to pinpoint the specific ergonomic risk factors causing WRMDs.

## Conclusion

This study reveals that work-related musculoskeletal disorders are highly prevalent among shopkeepers in Northwestern Ethiopia. The most frequently affected body part is the lower back, followed by the upper back, and shoulder. Being female, increasing age, being overweight, job stress, and sitting in the same position for two or more hours have been identified as significant factors for WRMSDs. To curb the onset of WRMSDs, contemporary workplace health and safety programs need to consider the diverse characteristics of the individuals, including gender, age, and BMI. Injury prevention efforts would also benefit from targeting occupational exposures, including static working postures and stress emanating from occupations.

## Acknowledgements

We would like to thank the University of Gondar, College of Medicine and Health Sciences and Comprehensive Specialized Hospital, Institute of Public Health for providing ethical clearance. The authors are also very much grateful to all the data collectors, the supervisor, and study participants.

## List of abbreviations

**AOR:** Adjusted Odds Ratios; **BMI:** Body Mass Index, **CI:** Confidence Interval; **COR:** Crude Odds Ratio, **ETB:** Ethiopian Birr, **Epi Info:** statistical software for epidemiology developed, **MSDs:** Musculoskeletal Disorders**, NMQ:** Nordic Musculoskeletal Questionnaire**, OR:** Odds Ratios; **STATA:** Statistical software for data science, **SDGs:** Sustainable Development Goals**,**

**WRMSDs:** Work-related Musculoskeletal Disorders, **YLDs:** Years Lived with Disability **Consent for publication**

Not applicable.

## Availability of data and materials

Authors presented the data in the main paper and made available from the corresponding author on a reasonable request via e-mail address: amensisahailu@gmail.com.

**Competing interests**

The authors declare that they have no competing interests.

## Funding

Not applicable since funding was not received from any agency in the public, commercial or notfor- profit sectors.

## Author Contributions

**Conceptualization:** Amensisa Hailu Tesfaye, Gebisa Guyasa Kabito,

**Data curation:** Amensisa Hailu Tesfaye, Gebisa Guyasa Kabito, Fantu Mamo Aragaw, and Tesfaye Hambisa Mekonnen

**Formal analysis:** Amensisa Hailu Tesfaye, Gebisa Guyasa Kabito, Fantu Mamo Aragaw, and Tesfaye Hambisa Mekonnen

**Investigation:** Amensisa Hailu Tesfaye, Gebisa Guyasa Kabito, Fantu Mamo Aragaw, and Tesfaye Hambisa Mekonnen

**Methodology:** Amensisa Hailu Tesfaye, Gebisa Guyasa Kabito, Fantu Mamo Aragaw, and Tesfaye Hambisa Mekonnen

**Software:** Amensisa Hailu Tesfaye

**Supervision:** Amensisa Hailu Tesfaye, Gebisa Guyasa Kabito, Fantu Mamo Aragaw, and Tesfaye Hambisa Mekonnen

**Validation:** Amensisa Hailu Tesfaye, Gebisa Guyasa Kabito, Fantu Mamo Aragaw, and Tesfaye Hambisa Mekonnen

**Writing-original draft:** Amensisa Hailu Tesfaye, Gebisa Guyasa Kabito, Fantu Mamo Aragaw, and Tesfaye Hambisa Mekonnen

**Writing-review and editing:** Amensisa Hailu Tesfaye, Gebisa Guyasa Kabito, Fantu Mamo

Aragaw, and Tesfaye Hambisa Mekonnen

## References

1. Executive H: **Work related Musculoskeletal Disorders (WRMSDs) Statistics in Great Britain.**

**Data up to March 2021. Annual statistics Published 16th December 2021//hse. gov. uk:[сайт]**. *URL: https://www hse gov uk/statistics/causdis/msd pdf (дата обращения: 2206 2021)*.

2. Bhandare A, Kulkarni A, Sanklecha S, Chitapure T: **Prevalence of Low Back Pain in Security Guards in MGM Institute of Health Sciences, Aurangabad**. *International Journal of Health Sciences and Research* 2020, **10**(9):336-345.
3. De Kok J, Vroonhof P, Snijders J, Roullis G, Clarke M, Peereboom K, Dorst P, Isusi I: **Workrelated MSDs: prevalence, costs and demographics in the EU**. *European Agency for Safety and Health at Work (EU-OSHA)* 2019.
4. Tesfaye AH, Mekonnen TH, Alemayehu M, Abere G: **Prevalence and Risk Factors of WorkRelated Upper Extremity Disorders among University Teaching Staff in Ethiopia, 2021: An Institution-Based Cross-Sectional Study**. *Pain Research and Management* 2022, **2022**.
5. Mekonnen TH, Kekeba GG, Azanaw J, Kabito GG: **Prevalence and healthcare seeking practice of work-related musculoskeletal disorders among informal sectors of hairdressers in Ethiopia, 2019: findings from a cross-sectional study**. *BMC Public Health* 2020, **20**(1):1-10.
6. Williams A, Kamper SJ, Wiggers JH, O’Brien KM, Lee H, Wolfenden L, Yoong SL, Robson E, McAuley JH, Hartvigsen J: **Musculoskeletal conditions may increase the risk of chronic disease: a systematic review and meta-analysis of cohort studies**. *BMC medicine* 2018, **16**(1):1-

9.

7. Briggs AM, Cross MJ, Hoy DG, Sanchez-Riera L, Blyth FM, Woolf AD, March L:

**Musculoskeletal health conditions represent a global threat to healthy aging: a report for the 2015 World Health Organization world report on ageing and health**. *The Gerontologist* 2016, **56**(suppl_2):S243-S255.

8. Woolf AD, Pfleger B: **Burden of major musculoskeletal conditions**. *Bulletin of the world health organization* 2003, **81**(9):646-656.
9. Crawford JO, Berkovic D, Erwin J, Copsey SM, Davis A, Giagloglou E, Yazdani A, Hartvigsen J, Graveling R, Woolf A: **Musculoskeletal health in the workplace**. *Best practice & research clinical rheumatology* 2020, **34**(5):101558.
10. Daneshmandi H, Choobineh A, Ghaem H, Alhamd M, Fakherpour A: **The effect of musculoskeletal problems on fatigue and productivity of office personnel: a cross-sectional study**. *Journal of preventive medicine and hygiene* 2017, **58**(3):E252.
11. Amell T, Kumar S: **Work-related musculoskeletal disorders: design as a prevention strategy. A review**. *Journal of occupational rehabilitation* 2001, **11**(4):255-265.
12. Govaerts R, Tassignon B, Ghillebert J, Serrien B, De Bock S, Ampe T, El Makrini I, Vanderborght B, Meeusen R, De Pauw K: **Prevalence and incidence of work-related musculoskeletal disorders in secondary industries of 21st century Europe: a systematic review and metaanalysis**. *BMC musculoskeletal disorders* 2021, **22**(1):1-30.
13. Waddell G: **Preventing incapacity in people with musculoskeletal disorders**. *British medical bulletin* 2006, **77**(1):55-69.
14. Woolf AD, Vos T, March L: **How to measure the impact of musculoskeletal conditions**. *Best Practice & Research Clinical Rheumatology* 2010, **24**(6):723-732.
15. Cieza A, Causey K, Kamenov K, Hanson SW, Chatterji S, Vos T: **Global estimates of the need for rehabilitation based on the Global Burden of Disease study 2019: a systematic analysis for the Global Burden of Disease Study 2019**. *The Lancet* 2020, **396**(10267):2006-2017.
16. Fink DL, Oladele D, Etomi O, Olaosebikan H, Dey ID, Adelowo OO: **We must harness technology to deliver the musculoskeletal disease epidemiology that is urgently needed across sub-Saharan Africa**. *Clinical rheumatology* 2018, **37**(5):1439-1440.
17. Woolf AD, Brooks P, Åkesson K, Mody GM: **Prevention of musculoskeletal conditions in the developing world**. *Best Practice & Research Clinical Rheumatology* 2008, **22**(4):759-772.
18. Badley E, Webster G, Rasooly I: **The impact of musculoskeletal disorders in the population:**

**are they just aches and pains? Findings from the 1990 Ontario Health Survey**. *The Journal of rheumatology* 1995, **22**(4):733-739.

19. Picavet H, Schouten J: **Musculoskeletal pain in the Netherlands: prevalences, consequences and risk groups, the DMC3-study**. *Pain* 2003, **102**(1-2):167-178.
20. Mekonnen TH, Yenealem DG, Geberu DM: **Physical environmental and occupational factors inducing work-related neck and shoulder pains among self-employed tailors of informal sectors in Ethiopia, 2019: results from a community based cross-sectional study**. *BMC Public Health* 2020, **20**(1):1-10.
21. Gupta M: **Study on Prevalence of Musculoskeletal Discomforts and Quality of Life in Among**

**Indian Shopkeepers Due to Chronic Osteoarthritic Pain**. *ARCHIVOS DE MEDICINA* 2021, **7**(7):145.

22. Hussain T, Taufiq F, ul Hassan T: **Prevalence and risk factors for low back pain among shopkeepers/salesman at model town link road Lahore, Pakistan**. *Rawal Medical Journal* 1970, **42**(2):162-162.
23. Wanyonyi N, Frantz J: **Prevalence of work-related musculoskeletal disorders in Africa: a systematic review**. *Physiotherapy* 2015, **101**:e1604-e1605.
24. Gebreyesus T, Nigussie K, Gashaw M, Janakiraman B: **The prevalence and risk factors of workrelated musculoskeletal disorders among adults in Ethiopia: a study protocol for extending a**

**systematic review with meta-analysis of observational studies**. *Systematic reviews* 2020, **9**(1):16.

25. Rahman A: **Work relatedmusculoskeletal disorders among the shopkeepers**. Bangladesh Health Professions Institute, Faculty of Medicine, the University …; 2015.
26. Nunes IL, Bush PM: **Work-related musculoskeletal disorders assessment and prevention**. *Ergonomics-A Systems Approach* 2012:1-30.
27. McCauley-Bush P: **Ergonomics: foundational principles, applications, and technologies**: CRC Press; 2011.
28. Etana G, Ayele M, Abdissa D, Gerbi A: **Prevalence of work related musculoskeletal disorders and associated factors among bank staff in Jimma city, Southwest Ethiopia, 2019: an institution-based cross-sectional study**. *Journal of Pain Research* 2021, **14**:2071.
29. Tegenu H, Gebrehiwot M, Azanaw J, Akalu TY: **Self-reported work-related musculoskeletal disorders and associated factors among restaurant workers in Gondar city, Northwest Ethiopia, 2020**. *Journal of Environmental and Public Health* 2021, **2021**.
30. Laal F, Madvari RF, Balarak D, Mohammadi M, Dortaj E, Khammar A, Adineh HA: **Relationship between musculoskeletal disorders and anthropometric indices among bus drivers in Zahedan city**. *International Journal of Occupational Safety and Ergonomics* 2018, **24**(3):431-437.
31. Chang K-C, Liao Y-H, Lee H-C, Wu C-Y, Yen C-L, Lin P-L, Hung J-W, Huang Y-C, Chou M-C, Li Y-H: **Musculoskeletal disorders, psychosocial stress and associated factors among homebased migrant care workers**. *Work* 2020, **65**(3):647-659.
32. Ababa A, Commission PC: **Summary and statistical report of the 2007 population and housing census. population size by age and sex**. 2008.
33. FDRo E: **Population projection of ethiopia for all regions, at wereda level from 2014–2017**. *Agency CS, editor Addis Ababa* 2013.
34. Leulseged T, Tsegaye MA: **The Role of Business Development Services in Micro and Small Enterprises Development (The Case of Gondar City Administration)**.
35. Daniel WW, Cross CL: **Biostatistics: a foundation for analysis in the health sciences**: Wiley; 2018.
36. Martínez-Mesa J, González-Chica DA, Bastos JL, Bonamigo RR, Duquia RP: **Sample size: how many participants do I need in my research?** *Anais brasileiros de dermatologia* 2014, **89**:609615.
37. Kuorinka I, Jonsson B, Kilbom A, Vinterberg H, Biering-Sørensen F, Andersson G, Jørgensen K: **Standardised Nordic questionnaires for the analysis of musculoskeletal symptoms**. *Applied ergonomics* 1987, **18**(3):233-237.
38. Von Korff M, Ormel J, Keefe FJ, Dworkin SF: **Grading the severity of chronic pain**. *Pain* 1992, **50**(2):133-149.
39. The Marlin Company NH, CT, and the American Institute of Stress, Yonkers, NY,: **The Workplace Stress Scale™** Available at: https://teorionline.files.wordpress.com/2011/04/unit-3the-workplace-stress-scale.pdf . Accessed on 10 June 2019.
40. Macdonald S, Maclntyre P: **The generic job satisfaction scale: Scale development and its correlates**. *Employee Assistance Quarterly* 1997, **13**(2):1-16.
41. Rolander B, Bellner A-L: **Experience of musculo-skeletal disorders, intensity of pain, and general conditions in work--the case of employees in non-private dental clinics in a county in southern Sweden**. *Work* 2001, **17**(1):65-73.
42. Nakata A, Ikeda T, Takahashi M, Haratani T, Hojou M, Swanson NG, Fujioka Y, Araki S: **The prevalence and correlates of occupational injuries in small****scale manufacturing enterprises**. *Journal of occupational health* 2006, **48**(5):366-376.
43. Gebremichael G, Kumie A: **The prevalence and associated factors of occupational injury among workers in Arba Minch textile factory, southern Ethiopia: a cross sectional study**. *Occupational medicine and health affairs* 2015, **3**(6):e1000222-e1000222.
44. Melchior M, Niedhammer I, Berkman L, Goldberg M: **Do psychosocial work factors and social relations exert independent effects on sickness absence? A six year prospective study of the GAZEL cohort**. *Journal of Epidemiology & Community Health* 2003, **57**(4):285-293.
45. Kee A: **Collusive Pricing under the Commerce Act 1975**. *Victoria U Wellington L Rev* 1984, **14**:183.
46. Kunda R, Frantz J, Karachi F: **Prevalence and ergonomic risk factors of work-related musculoskeletal injuries amongst underground mine workers in Zambia**. *Journal of occupational health* 2013, **55**(3):211-217.
47. Hossain MD, Aftab A, Al Imam MH, Mahmud I, Chowdhury IA, Kabir RI, Sarker M: **Prevalence of work related musculoskeletal disorders (WMSDs) and ergonomic risk assessment among readymade garment workers of Bangladesh: A cross sectional study**. *PloS one* 2018, **13**(7):e0200122.
48. Gupta G, Bhavana R, Rishikesh R: **Reliability and validity of Hindi version of Nordic musculoskeletal questionnaire**. *EC Orthop* 2018, **9**(8):533-538.
49. Chairani A: **Validity and reliability test of the Nordic Musculoskeletal questionnaire with formal and informal sector workers**. In: *7th International Conference on Public Health 2020: 2020*: Sebelas Maret University; 2020: 100-106.
50. Merkeb Alamneh Y, Sume BW, Abebaw Shiferaw A: **Musculoskeletal disorders among the population in Northwest Ethiopia**. *SAGE open medicine* 2022, **10**:20503121221085109.
51. Hosmer DW, Hjort NL: **Goodness****of****fit processes for logistic regression: simulation results**. *Statistics in medicine* 2002, **21**(18):2723-2738.
52. Kasaw Kibret A, Fisseha Gebremeskel B, Embaye Gezae K, Solomon Tsegay G: **Work-related musculoskeletal disorders and associated factors among bankers in Ethiopia, 2018**. *Pain Research and Management* 2020, **2020**.
53. Abledu J, Abledu G: **Multiple logistic regression analysis of predictors of musculoskeletal disorders and disability among bank workers in Kumasi**. *Ghana J Ergonomics* 2012, **2**(111):2.
54. Mishra S, Sarkar K: **Work-related musculoskeletal disorders and associated risk factors among urban metropolitan hairdressers in India**. *Journal of occupational health* 2021, **63**(1):e12200.
55. Yang S, Lu J, Zeng J, Wang L, Li Y: **Prevalence and risk factors of work-related musculoskeletal disorders among intensive care unit nurses in China**. *Workplace health & safety* 2019, **67**(6):275-287.
56. Côté JN: **A critical review on physical factors and functional characteristics that may explain a sex/gender difference in work-related neck/shoulder disorders**. *Ergonomics* 2012, **55**(2):173182.
57. Haizlip K, Harrison B, Leinwand L: **Sex-based differences in skeletal muscle kinetics and fibertype composition**. *Physiology* 2015, **30**(1):30-39.
58. Messing K, Punnett L, Bond M, Alexanderson K, Pyle J, Zahm S, Wegman D, Stock SR, de Grosbois S: **Be the fairest of them all: challenges and recommendations for the treatment of gender in occupational health research**. *American journal of industrial medicine* 2003, **43**(6):618-629.
59. Mekonnen TH: **The magnitude and factors associated with work-related back and lower extremity musculoskeletal disorders among barbers in Gondar town, northwest Ethiopia, 2017: A cross-sectional study**. *Plos one* 2019, **14**(7):e0220035.
60. Heidari M, Borujeni MG, Rezaei P, Abyaneh SK: **Work-related musculoskeletal disorders and their associated factors in nurses: A cross-sectional study in iran**. *The Malaysian journal of medical sciences: MJMS* 2019, **26**(2):122.
61. Freemont A, Hoyland J: **Morphology, mechanisms and pathology of musculoskeletal ageing**. *The Journal of Pathology: A Journal of the Pathological Society of Great Britain and Ireland* 2007, **211**(2):252-259.
62. Cassou B, Derriennic F, Monfort C, Norton J, Touranchet A: **Chronic neck and shoulder pain, age, and working conditions: longitudinal results from a large random sample in France**. *Occupational and environmental medicine* 2002, **59**(8):537-544.
63. de Zwart BC, Frings-Dresen MH, Van Dijk FJ: **Physical workload and the ageing worker: a review of the literature**. *International archives of occupational and environmental health* 1996, **68**(1):1-12.
64. Mohan P, Sudhan S, Dhasaradharaman K, Suresh A: **Work-related musculoskeletal injury risk assessment and its relevance to body mass index for computer professionals**. *Biomedical and Pharmacology Journal* 2020, **13**(2):843-848.
65. Sethi J, Sandhu JS, Imbanathan V: **Effect of Body Mass Index on work related musculoskeletal discomfort and occupational stress of computer workers in a developed ergonomic setup**. *Sports Medicine, Arthroscopy, Rehabilitation, Therapy & Technology* 2011, **3**(1):1-7.
66. Hutabarat J: **The Relation of Musculoskeletal Discomfort with Body Mass Index (BMI) For Cleaning Workers, Who Work at an Elevated Place**. *International Journal of Engineering Research and Technology* 2020, **3**(5):938-942.
67. Park W, Ramachandran J, Weisman P, Jung ES: **Obesity effect on male active joint range of motion**. *Ergonomics* 2010, **53**(1):102-108.
68. Viester L, Verhagen EA, Hengel KMO, Koppes LL, van der Beek AJ, Bongers PM: **The relation between body mass index and musculoskeletal symptoms in the working population**. *BMC musculoskeletal disorders* 2013, **14**(1):1-9.
69. Dagne D, Abebe SM, Getachew A: **Work-related musculoskeletal disorders and associated factors among bank workers in Addis Ababa, Ethiopia: a cross-sectional study**. *Environmental health and preventive medicine* 2020, **25**(1):1-8.
70. Baxi MS, Hande DN: **Assessment of ergonomic risk factor and functional status among bank workers of loni**. *Imperial Journal of Interdisciplinary Research (IJIR)* 2017, **1**(2):2454-1362.
71. Sulaiman SK, Kamalanathan P, Ibrahim AA, Nuhu JM: **Musculoskeletal disorders and associated disabilities among bank workers**. *Int J Res Med Sci* 2015, **3**(5):1153-1158.
72. Johnson EO, Kamilaris TC, Chrousos GP, Gold PW: **Mechanisms of stress: a dynamic overview of hormonal and behavioral homeostasis**. *Neuroscience & Biobehavioral Reviews* 1992, **16**(2):115-130.
73. Nagalski A, Kiersztan A: **Fizjologia i molekularny mechanizm działania glikokortykoidów**

**[Physiology and molecular mechanism of glucocorticoidaction]**. *Postepy Hig Med Dosw (Online)* 2010, **64**:133-145.

74. Pruszkowska-Przybylska P, Sitek A, Rosset I, Sobalska-Kwapis M, Słomka M, Strapagiel D, Żądzińska E, Morling N: **Cortisol concentration affects fat and muscle mass among Polish children aged 6–13 years**. *BMC pediatrics* 2021, **21**(1):1-8.
75. PRESS NA, Washington DC: **Musculoskeletal Disorders and the Workplace: Low Back and Upper Extremities** 2001(Musculoskeletal Disorders and the Workplace: Low Back and Upper Extremities):512.

730 76. de Langen N, Peereboom K: **Musculoskeletal disorders and prolonged static sitting**. *OSHwiki,* 731 *2020a Available at: https://oshwiki*

*eu/wiki/Musculoskeletal_disorders_and_prolonged_static_sitting* 2020.

732

733

734

735 736

737

738

739

740

741

742

743

744

745

746

747

748

749

750

751

752

753

754

755

756 77.

78.

Dennison MS, D’Zmura M: **Cybersickness without the wobble: Experimental results speak against postural instability theory**. *Applied ergonomics* 2017, **58**:215-223.

Naveed N, Kannan M: **MUSCULOSKELETAL DISORDERS IN DENTISTRY**.

## 757 List of tables

758 **Table 1:** Socio-demographic characteristics of shopkeepers in Northwest Ethiopia, 2022 (N=625).

| **Variables** | **Frequency** | **Percent (%)** |
| --- | --- | --- |
| **Sex** |  |  |
| Male | 321 | 51.4 |
| Female | 304 | 48.6 |
| **Religion** |  |  |
| Orthodox | 338 | 54.1 |
| Muslim | 178 | 28.5 |
| Protestant | 94 | 15.0 |
| Catholic | 15 | 2.4 |
| **Age in years** |  |  |
| 20-29 | 196 | 31.40 |
| 30-39 | 228 | 36.50 |
| ≥40 | 201 | 32.10 |
| **Marital status** |  |  |
| Single | 178 | 28.5 |
| Married | 375 | 60.0 |
| Divorced | 34 | 5.4 |
| Widowed | 38 | 6.1 |
| **Educational level** |  |  |
| Unable to read and write | 13 | 2.1 |
| Primary school complete | 250 | 40.0 |
| Secondary school complete | 265 | 42.4 |
| Certificate/diploma/degree | 97 | 15.5 |
| **Work experience in current shop (years)** |  |  |
| 1-5 | 304 | 48.6 |
| 6-10 | 223 | 35.7 |
| >10 | 98 | 15.7 |
| **Monthly salary (ETB)** |  |  |
| <4000 | 218 | 34.9 |
| ≥4000 | 407 | 65.1 |
| **Family size in person** |  |  |
| <5 | 209 | 33.4 |
| ≥5 | 416 | 66.6 |

759 **Key:** ETB= Ethiopian Birr (currency)

**Table 2:** Behavioral and psychosocial characteristics of shopkeepers in Northwest Ethiopia, 2022 (N=625).

| **Variables** | **Frequency** | **Percent (%)** |
| --- | --- | --- |
| **Body mass index (BMI)** |  |  |
| Underweight | 31 | 5.0 |
| Normal | 443 | 70.9 |
| Overweight | 151 | 24.2 |
| **Physical exercise** |  |  |
| Yes | 73 | 11.7 |
| No | 552 | 88.3 |
| **Cigarette smoke** |  |  |
| Yes | 56 | 9.0 |
| No | 569 | 91.0 |
| **Drink alcohol** |  |  |
| Yes | 181 | 29.0 |
| No | 444 | 71.0 |
| **Khat chawing behavior** |  |  |
| Yes | 48 | 7.7 |
| No | 577 | 92.3 |
| **Chronic illness** |  |  |
| Yes | 80 | 12.8 |
| No | 545 | 87.2 |
| **Chronic illness reported** |  |  |
| Asthma | 26 | 4.2 |
| Hypertension | 15 | 2.4 |
| Diabetes | 33 | 5.3 |
| Others**^#^** | 6 | 1.0 |
| **Perceived musculoskeletal pain severity** |  |  |
| High | 127 | 20.3 |
| Low | 498 | 79.7 |
| **Perceived musculoskeletal pain disability** |  |  |
| High | 160 | 25.6 |
| Low | 465 | 74.4 |
| **Perceived job satisfaction** |  |  |
| Satisfied | 452 | 72.3 |
| Not satisfied | 173 | 27.7 |
| **Perceived job stress** |  |  |
| Stressed | 401 | 64.2 |
| Not stressed | 224 | 35.8 |

^#^Heart disease, Kidney stone,

763	**Table 3:** Work-environment and ergonomic characteristics of shopkeepers in Northwest Ethiopia, 764	2022 (N=625).

| **Variables** | **Frequency** | **Percent (%)** |
| --- | --- | --- |
| **Work place (shop) location** |  |  |
| Ground | 528 | 84.5 |
| First floor | 62 | 9.9 |
| Second floor and above | 35 | 5.6 |
| **Type of shop** |  |  |
| Supermarket | 35 | 5.6 |
| Clothe, shoe, and bag shop | 162 | 25.9 |
| Small local shop (wholesalers) | 192 | 30.7 |
| Stationery and book store shop | 64 | 10.2 |
| Electronics and hardware shop | 97 | 15.5 |
| Greengrocer (fruit and vegetable) shop | 56 | 9.0 |
| Others^b^ | 19 | 3.0 |
| **Working hours per day** |  |  |
| ≤8hrs | 178 | 28.5 |
| >8hrs | 447 | 71.5 |
| **Total working day per week** |  |  |
| ≤5 days/week | 279 | 44.6 |
| >5 day/week | 346 | 55.4 |
| **Awkward postures** |  |  |
| Yes | 387 | 61.9 |
| No | 238 | 38.1 |
| **Taking rest breaks** |  |  |
| Yes | 186 | 29.8 |
| No | 439 | 70.2 |
| **Types of sitting chair** |  |  |
| Fixed | 578 | 92.5 |
| Adjustable | 47 | 7.5 |
| **Prolonged standing** |  |  |
| Yes | 391 | 62.6 |
| No | 234 | 37.4 |

| **Prolonged sitting** |  |  |
| --- | --- | --- |
| Yes | 260 | 41.6 |
| No | 365 | 58.4 |
| **Repetitive movement** |  |  |
| Yes | 393 | 62.9 |
| No | 232 | 37.1 |
| **Lifting or carrying heavy loads** |  |  |
| Yes | 369 | 59.0 |
| No | 256 | 41.0 |
| **Climbing stairs or ladders** |  |  |
| Yes | 328 | 52.5 |
| No | 297 | 47.5 |

^b^souvenir, jeweler, and cosmetics shop

**Table 4:** Association between one-year cumulative prevalence of work-related musculoskeletal disorders and sex differences among shopkeepers (N=625).

| **Sex** | **work-related MSDs** | | **χ2 value** | **p-value** | **CI (95%)** | **AOR** |
| --- | --- | --- | --- | --- | --- | --- |
|  | **Yes** | **No** |  |  |  |  |
| Female | 257 (84.5%) | 47 (15.5%) | 4.5189 | 0.002* | 1.30 - 3.30 | 2.07 |
| Male | 250 (77.9%) | 71 (22.1%) |  |  |  |  |

Chi-square Test

*Significant p-value < 0.05

**Table 5:** The 12 months prevalence of work-related musculoskeletal disorders by body region (N=625).

| **Work-related musculoskeletal disorders** | | **Male** | **Female** | **χ2 value** | **p-value** |
| --- | --- | --- | --- | --- | --- |
|  |  | **Frequency (%)** | **Frequency (%)** |  |  |
| Neck | Yes | 48 (15%) | 50 (16.4%) | 0.2636 | 0.608 |
|  | No | 273 (85%) | 254 (83.6%) |  |  |
| Shoulder | Yes | 110 (34.3%) | 111 (36.5%) | 0.3444 | 0.557 |
|  | No | 211 (65.7%) | 193 (63.5%) |  |  |
| Upper back | Yes | 115 (35.8%) | 167 (54.9%) | 16.6823 | **0.000*** |
|  | No | 206 (64.2%) | 137 (45.1%) |  |  |
| Elbow | Yes | 69 (21.5%) | 61 (20.1%) | 0.1937 | 0.660 |
|  | No | 252 (78.5%) | 243 (79.9%) |  |  |
| Lower back | Yes | 124 (38.6%) | 167 (54.9%) | 16.6823 | **0.000*** |
|  | No | 197 (61.4%) | 137 (45.1%) |  |  |
| Hand/wrist | Yes | 75 (23.4%) | 70 (23.0%) | 0.0100 | 0.920 |
|  | No | 246 (76.6%) | 234 (77.0%) |  |  |
| Hip/thigh | Yes | 63 (19.6%) | 67 (22.0%) | 0.5520 | 0.458 |
|  | No | 258 (80.4%) | 237 (78.0%) |  |  |
| Knee | Yes | 110 (34.3%) | 90 (29.6%) | 1.5599 | 0.212 |
|  | No | 211 (65.7%) | 214 (70.4%) |  |  |
| Ankle/foot | Yes | 74 (23.1%) | 73 (24.0%) | 0.0800 | 0.777 |
|  | No | 247 (76.9%) | 231 (76.0%) |  |  |

Chi-square Test

*Significant p-value < 0.001

787 **Table 6**: Multi-variable analysis of factors associated with work-related musculoskeletal disorders 788 among shopkeepers in Northwest Ethiopia, 2022 (N= 625).

| **Variables** | **WRMSDs** | | **COR (95% CI)** | **P-value** | **AOR (95% CI)** | **P-value** |
| --- | --- | --- | --- | --- | --- | --- |
|  | **Yes** | **No** |  |  |  |  |
| **Sex** | | |  |  |  |  |
| Male | 250 | 71 | 1 |  | 1 |  |
| Female | 257 | 47 | 1.55 (1.03-2.33) | 0.034 | **2.09 (1.31-3.33)** | **0.002*** |
| **Age** | | |  |  |  |  |
| 20-29 | 139 | 57 | | 1 |  |  |
| 30-39 | 187 | 41  | | 0.007 | **1.92 (1.15-3.21)** | **0.013** |
| ≥40 | 181 | 20 | | 0.000 | **3.86 (2.06-7.29)** | **0.000**** |
| **Work experience in years** | | |  |  |  |  |
| 1-5 | 240 | 64 | 1 |  | 1 |  |
| 6-10 | 181 | 42 | 1.15 (0.74-1.77) | 0.530 | 1.44 (0.88-2.35) | 0.148 |
| >10 | 86 | 12 | 1.91 (0.98-3.71) | 0.056 | 1.56 (0.74-3.31) | 0.246 |
| **Family size** | | |  |  |  |  |
| <5 | 163 | 46 | 1 |  | 1 |  |
| ≥5 | 344 | 72 | 1.35 (0.89-2.04) | 0.157 | 1.28 (0.80-2.10) | 0.305 |
| **Body mass index (BMI)** | | |  |  |  |  |
| Underweight | 20 | 11 | 0.48 (0.22-1.03) | 0.059 | 0.72 (0.31-1.75) | 0.456 |
| Overweight | 136 | 15 | 2.38 (1.33-4.25) | 0.003 | **2.04 (1.10-3.82)** | **0.025*** |
| Normal | 351 | 92 | 1 |  | 1 |  |
| **Chronic illness** |  |  |  |  |  |  |
| Yes | 70 | 10 | 1.73 (0.86-3.47) | 0.122 | 1.46 (0.47-4.48) | 0.511 |
| No | 437 | 108 | 1 |  | 1 |  |
| **Perceived job stress** | | |  |  |  |  |
| Stressed | 361 | 40 | 4.82 (3.15-7.39) | 0.000 | **4.09 (2.61-6.43)** | **0.000**** |
| Not stressed | 146 | 78 | 1 |  | 1 |  |
| **Perceived musculoskeletal pain severity** | | |  |  |  |  |
| High | 109 | 18 | 1.52 (0.88-2.62) | 0.131 | 1.37 (0.57-3.27) | 0.481 |
| Low | 398 | 100 | 1 |  | 1 |  |
| **Prolonged sitting** | | |  |  |  |  |
| Yes | 224 | 36 | 1.80 (1.17-2.77) | 0.007 | **1.70 (1.06-2.74)** | **0.028 *** |
| No | 283 | 82 | 1 |  | 1 |  |

**Keys:** 1=reference category, AOR=adjusted odds ratio, CI= confidence interval, COR=crudes odds ratio, WRMSDs=work-related musculoskeletal disorders, *= significant at p-value < 0.05, **Significant at p-value < 0.001 in multivariable logistic regression analysis, Hosmer-Lemeshow test (HL test) p-value = 0.5491.

Figure	[Click here to access/download;Figure;List of Figures](https://www.editorialmanager.com/pone/download.aspx?id=32133345&guid=d8bc4fc3-24fa-4faa-91ed-99b144266050&scheme=1)

[shopkeepers.docx](https://www.editorialmanager.com/pone/download.aspx?id=32133345&guid=d8bc4fc3-24fa-4faa-91ed-99b144266050&scheme=1)

## List of figures

**Figure 1:** Schematic representation of sampling procedure.

**Figure 2:** Prevalence of work-related musculoskeletal disorders by body regions among shopkeepers in the last 12 months.

**Figure 3:** Prevalence of work-related musculoskeletal disorders by body regions among shopkeepers in the last 7 days.

## Figure 1

Arada sub city

N=3459 shops

Maraki sub city

N=2559 shops

Proportional allocation

Maraki

sub city

N=270 shops

Arada sub city

N=364 shops

**Gondar city**

Sub cities = 6

Systematic random sampling

Lottery method

Total

N

=634

## Figure 2

## Figure 3
