# Supplementary material for: Prevalence and risk factors of work-related musculoskeletal disorders among shopkeepers in Ethiopia: Evidence from a workplace cross-sectional study
Source: PLoS One. 2024 Mar 21;19(3):e0300934. doi: 10.1371/journal.pone.0300934 (PMC10956884; doi:10.1371/journal.pone.0300934)
Supplement: Attachment — Submitted filename: Response to Reviewers.docx [file pone.0300934.s005.docx]

**Date:** 19 November 2023

**To**: PLOS ONE, Editorial manager

**Subject**: - Point-by-point response to the reviewer’s comments (Manuscript ID**:** **PONE-D-22-30367R1**)

**Title**: Prevalence and risk factors of work-related musculoskeletal disorders among shopkeepers in Northwestern Ethiopia: evidence from a workplace cross-sectional study

**Dear Editorial manager,**

We are very grateful for receiving the reviewers’ comments on our manuscript, which we believe will improve our manuscript. Following the reviewers' valuable comments and recommendations, we have revised the work, and we hereby submit the revised work for your consideration along with a point-by-point response to the reviewers.

Kindest regards,

Authors

**A point-by-point response to Editor and Reviewers**

Thank you all for your constructive and valuable comments and concerns.

Note that all changes to the manuscript are included in the track change too.

**Title**: Prevalence and risk factors of work-related musculoskeletal disorders among shopkeepers in Northwestern Ethiopia: evidence from a workplace cross-sectional study

**Date:** 19 November 2023

**Reviewer #3:** Abebaw Jember Ferede

Responses to Reviewer #3 *(****Reference****s correspond to* ***cleaned (revised) version manuscript****)*

| Dear Abebaw, thank you for taking the time to provide useful and important comments to help us improve our manuscripts before publication. We all authors accepted and acknowledged your comments. You have given the comments in a modified truck format, which is very much appreciated by all the authors. We feel that many of your comments have been addressed in our previously revised and summited version of the manuscript (revision one). You can view the previously revised manuscript with its tracked changed format. We apologize if you have not had access to the previously revised and edited version of our summitted manuscript.  We have given time for your comments and extensively gone through them to revise our current manuscript, since we found your comments to be very important for the improvements of the manuscript. Now, hopefully, you are very interested in our revised version manuscript. Moreover, we have provided the following point-by-point response to your comments and suggestions. | | |
| --- | --- | --- |
| **Question/comment** | **Responses** | **Reference** |
| Please replace the AOR and CI with p-value. | Thank you for your comment. The AOR and CI show both the direction and the strength of the association of the factors, but the p-value only shows the significance of the association. So, reporting AOR with CI is more important. In addition, reporting AOR and CI provides important information for other researchers who will do systematic and meta-analysis in this area. They can easily get the information they need from the abstract without reading the whole article. |  |
| Write either the American or British English, it is not advised to use both in a document. | Thank you very much for your acceptable comment. We have now revised and modified the manuscript accordingly. | See the cleaned-up manuscript, line 81, page 3 |
| Put such findings in 10th decimal points, as 56.3%, in a similar way in the document. | Thank you for raising this point. Following your comment, we have now corrected it | See the cleaned-up manuscript, line 85 |
| Please write the prevalence from global to local in a paragraph in a concise way. | Thank you for your comments. Your concern was absolutely correct. We have now modified it accordingly. All authors are grateful for all comments received, which helped to improve our revised manuscript for publication. | See the cleaned-up manuscript |
| How did you consider exercise related to their work, such as moving boxes, walking upstairs, etc.? | We measure physical exercise as someone who exercising or doing any kind of sports activity at least two times per week with a duration of at least 30 minutes. Moving boxes, walking upstairs, etc. are normal activities for our study population. The working nature of our study population includes such similar activities. We made this clear in the introduction. We also included these factors as factors of WRMSD, but in our results they were not significantly associated with WRMSD. |  |
| Alcohol drinking should also be measured in bottles. | Your comment is correct. Local drinks such as tella, tej, areki, etc. challenge us to measure them with a bottle, as they are drunk with local utensils. |  |
| Did you modify the Nordic Musculoskeletal Questionnaire, If so, how? | We have not modified it. It is a typo error; we have now corrected it accordingly |  |
| How was the questionnaire translated?  Who translated the questionnaire? | Thank you for your concern. We have clearly stated this issue in **data quality control section.** All authors acknowledge your comments and suggestions. | See the cleaned-up manuscript, lines 238-244, page 9 |
| Domains or sub-scales are parts of an instrument to measure a concept. Socio-demographic variables are not considered as a domain. | Thank you so much for your fruitful comments. We all agreed that the comment was correct, and we have now revised and corrected as per the comment. | See the cleaned-up manuscript, pages 7-9 |
| The CI can tell us if the AOR is significant or not, therefore, remove the p-values in the text and present it in the regression table. | Thank you for your comment. Your comment is accepted. Now we have removed the p-values from the text | See the cleaned-up manuscript, line 259 to 268 |

**Reviewer #4: Dr. Hardeep Rai Sharma, Institute of Environmental Studies, Kurukshetra University, Kurukshetra, Haryana, India**

*Responses to Reviewer #4 (****Reference****s correspond to* ***cleaned (revised) version manuscript****)*

| **Question/comment** | **Responses** | **Reference** |
| --- | --- | --- |
| The revised version of research paper entitled “Prevalence and risk factors of work-related musculoskeletal disorders among shopkeepers in Northwestern Ethiopia: evidence from a workplace cross-sectional study” is a good work conducted by the authors. The manuscript needs to incorporate the following comments/suggestions as: | Thank you very much for reviewing our manuscript and providing interesting comments to improve the manuscript. We have taken the time to consider your comments and have gone through them thoroughly in order to revise our manuscript, as we all authors accept that all comments are very important for the improvement our manuscript for publication. We hope that you are now interested in our revised version manuscript. | See the cleaned-up manuscript |
| 1. In the text author must quote reference carefully, e.g. in lines 102-103, after reference number 26, the next reference number will be 27 not 31? Authors are suggested to re-numbered the references accordingly both in main text and in reference section. | Thank you so much for your comment. The order of the reference is correct. Reference number 27 was found in the range of reference numbers 26-30. Reference numbers 4, 26 and 31, but not 27, support psychosocial factors associated with WRMSD. |  |
| 2. In line 146, write as “Maraki and Arada”. | Thank you for your comment. The comment has been accepted by all authors. We have now rewritten Maraki & and Arada as "Maraki and Arada" accordingly. | See the cleaned-up manuscript, line 146 |
| 3. In line 172, write as “Alcohol intake”. | Thank you so much for your comments. The authors think the comment is absolute. We have now rewrite it accordingly. | See the cleaned-up manuscript, line 172 |
| 4. Rewrite the lines 305-306, are authors explaining their results? If yes the how their results are in accordance with WHO classification? Also add reference support of WHO classification. | Thank you for your accurate comment. The authors are grateful for your comments. We have now succinctly revised and modified it following to your comments. | See the cleaned-up manuscript, lines 294-295 |
| 5. In lines 352 and 353 in kg/m2, “2” will be in superscript font. | The authors are grateful for your comments. Now we have written correctly in kg/m2, "2" in superscript. | See the cleaned-up manuscript, line 341 |
| 6. In reference section, line 518, 2022 appears twice. | In this reference section, the volume of the article is Volume 2022 and the article is published in 2022. Therefore, the reason 2022 appears twice is that. For more information, you can access the article and have a look at it. |  |
| 7. In lines 531 and 532 w, h and o in world health organization will be in capital font. | Dear Dr. Hardeep Rai Sharma, thank you for raising this point. We have now capitalized each letter (w, h and o) accordingly. |  |
| 8. Authors must write the references as per “PLOS one” style. | Thank you for your recommendation. We have accepted your comment and corrected the references as per “PLOS one” style. | See the cleaned-up manuscript |
| 9. Reference no 25 is not complete. | Thank you for your insightful comment and suggestion. The comment has been accepted. We have now completed the incomplete reference. | See the cleaned-up manuscript |
| 10. Add page no in reference no 33. | We have now added the page number in reference number 33 as per your insightful comment. |  |
| 11. In line 605, year 2020 appears twice. | In this reference section, the volume of the article is Volume 2020 and the article is published in 2020. Therefore, the reason 2020 appears twice is that. For more information, you can access the article and have a look at it. Thank you very much for your comment | See the cleaned-up manuscript |
| 12. Reference no 83 is not complete. | Thank you for your comment. All authors agreed that the comment was correct. Accordingly, we have now revised and completed the reference. | See the cleaned-up manuscript |
| 14. In Table 2 check the total of percentage of BMI and Chronic illness reported, it’s not 100 %? | Dear reviewer, Thank you so much for your interesting and thoughtful comments. It was a topographical error of original summitted manuscript. All the authors would like to thank you for all your important comments and suggestions, which helped to improve our revised manuscript for publication. | See the cleaned-up manuscript |

**We gratefully acknowledge every one of your insightful and incredibly intriguing remarks.**

**We look forward to your positive response as soon as possible.**

With best regards,

Sincerely yours,

Authors
