# Supplementary material for: Prevalence and risk factors of work-related musculoskeletal disorders among shopkeepers in Ethiopia: Evidence from a workplace cross-sectional study
Source: PLoS One. 2024 Mar 21;19(3):e0300934. doi: 10.1371/journal.pone.0300934 (PMC10956884; doi:10.1371/journal.pone.0300934)
Supplement: Attachment — Submitted filename: Comments_PLoS.doc [file pone.0300934.s006.doc]

03.12.2023

The re-revised version of the research paper entitled “Prevalence and risk factors of work-related musculoskeletal disorders among shopkeepers in Northwestern Ethiopia: evidence from a workplace cross-sectional study” is an appreciable efforts of the authors. Still, the manuscript needs to incorporate the following comments/suggestions as:

1. As suggested earlier, authors must write the reference carefully and strictly as per “PLOS ONE” style, still many references in the reference section needs careful editing e.g. reference number 8 (correct world health organization); no. 9-13, 16, 18, 24, 36-38, 43, 44, 47-49, 52, 53, 55, 59, 60, 63-65, 67, 68, 73, 74, 79 and 82 (write journal names properly); no. 15 (correct Burden of Disease); no. 80 (needs proper quoting) etc. Further many journal names are written full while other in abbreviations? Ref. no 25, 32, 80 and 83 are not complete add either their websites or other details. In ref no. 51 add country name after Sebelas Maret University. Authors are suggested to do changes in different font color in the revised R3 manuscript.
2. As suggested earlier, In Table 1 instead of using word “Sex” better to use “Gender”, what is authors opinion about the change?
3. The overall sample size for this study was 634 (lines 144 and Table 1) but authors analyzed 625 questionnaires (lines 288) what was the reason for 98.6% response rate can be briefly added in Socio-demographic characteristics of study participants section.
4. Author’s recommendations in lines 446-450 are OK but some sort of intervention or solution can be expected from Environmental and Occupational and Physiotherapy departments in the College of Medicine and Health Science of the University of Gondar, Ethiopia! Authors can suggests some basic exercises and correct sitting postures to the studied shopkeepers for a time period so that the purpose of “Community Service” for health professionals can be achieved.
