# Supplementary material for: Prevalence and risk factors of work-related musculoskeletal disorders among shopkeepers in Ethiopia: Evidence from a workplace cross-sectional study
Source: PLoS One. 2024 Mar 21;19(3):e0300934. doi: 10.1371/journal.pone.0300934 (PMC10956884; doi:10.1371/journal.pone.0300934)
Supplement: Attachment — Submitted filename: Response to reviewers.docx [file pone.0300934.s008.docx]

**Date:** 14 January 2024

**To**: PLOS ONE, Editorial manager

**Subject**: - Point-by-point response to the editor’s and reviewer’s comments (Manuscript ID**: PONE-D-22-30367R2**)

**Title**: Prevalence and risk factors of work-related musculoskeletal disorders among shopkeepers in Northwestern Ethiopia: evidence from a workplace cross-sectional study

**Dear Editorial manager,**

We are very grateful for receiving the editor’s and reviewers’ comments on our manuscript, which we believe will improve our manuscript. Following the reviewers' and editors' valuable comments and recommendations, we have revised the work, and we hereby submit the revised work for your consideration along with a point-by-point response to the editor and reviewers.

Kindest regards,

Authors

**A point-by-point response to Editor and Reviewers**

**Thank you all for your constructive and valuable comments and concerns.**

**Note that all changes to the manuscript are included in the track change too.**

**Title**: Prevalence and risk factors of work-related musculoskeletal disorders among shopkeepers in Northwestern Ethiopia: evidence from a workplace cross-sectional study

**Date:** 14 January 2024

**Reviewer: 3**

*Responses to the reviewer:3 (****Reference****s correspond to* ***cleaned (revised) version manuscript****)*

| **Comments** | **Responses** | **References** |
| --- | --- | --- |
| Gondar city, Ethiopia: Evidence…  The study is conducted in Gondar. I think it would be inappropriate to use northwest in the title as this is a large region that Gondar city is situated. | Thank you for your comment. We have accepted the comment and have corrected it accordingly. | See the cleaned-up manuscript (R3). |
| Avoid use of abbreviations in the abstract. | We have avoided using abbreviations in the abstract. | See the cleaned-up manuscript (R3). |
| Remove this from the abstract too. The findings should be presented without the PR, CI and other estimates. Only showing the significant variables with the corresponding regression coefficients or Betas and P-values is enough. | Thank you so much for your comments. We have now removed PR, CI from the abstract section and presented the significant variable solely with p-values. | See the cleaned-up manuscript (R3). |
| Please use p-values to show significant variables. The other details can be found in the body of the manuscript. | The comment has been accepted and corrected accordingly. | See the cleaned-up manuscript (R3). |
| This is a repetition of the results section as the significant variables are mentioned above there. Please indicate what this finding indicates in conclusion; what does this mean? | Thank you for your insightful comments. We have re-clarified the conclusion in the abstract section. |  |
| Low-income or low- and middle-income countries? Use the above terms to indicate as appropriate. | The comment has been accepted and corrected accordingly. | See the cleaned-up manuscript (R3). Line 59 |
| Does this mean quality of life? It would be better if it is described as quality of life. | Following your suggestion, the comment has been corrected. | See the cleaned-up manuscript (R3). |
| In this section of the paragraph global and worldwide are used unnecessarily. Please improve the quality of the manuscript by editing the language. | Thank you for your comments. We have edited and improved the manuscript accordingly. | See the cleaned-up manuscript (R3). |
| Studies or a study? How many studies support this evidence? | Sorry for the typo. It is a study. We have corrected it accordingly. | See the cleaned-up manuscript (R3). |
| Use similar format in presenting percentages, numbers after decimals, comparing this with the previous sentence 56.3%. | Comment has been accepted and corrected |  |
| Remove this as it is repeated below. Write as:  Where: n = initial sample size  Zα/2 = P = | Thank you for your comment. We have now corrected/rewrite it accordingly | See the cleaned-up manuscript (R3). |
| Who prepared the questionnaire? | The questionnaire was first prepared in English by the investigators and then translated into Amharic and back into English by language experts and physiotherapists to ensure consistency. |  |
| Justify why the analysis is changed to Poisson regression model? Of course, in the previous version of the manuscript, the data analysis method was multivariate logistic regression and that was also not right. Please make sure you use appropriate analysis method, binary logistic and multiple logistic regression model. | Thank you for raising this issue. As we reported a prevalence of 81.1%, it would have been good if they had used log binomial and reported the prevalence ratio instead of the odds ratio. The OR overestimates the PR and the way we interpreted the odds is not correct. So, we need to do a reanalysis using log binomial and report the prevalence ratio as the effect size measure. We have cited the article to support this. | See the cleaned-up manuscript (R3). |
|  | Other reviewer comments accepted and corrected accordingly.  All authors gratefully acknowledge your crucial comments. We have learnt a lot from your absolute comment. | See the cleaned-up manuscript (R3). |

**Reviewer: 4**

#### Responses to the reviewer:4 (References correspond to cleaned (revised) version manuscript)

| **Question/comment** | **Responses** | **Reference** |
| --- | --- | --- |
| 1. As suggested earlier, authors must write the reference carefully and strictly as per “PLOS ONE” style, still many references in the reference section needs careful editing e.g. reference number 8 (correct world health organization); no. 9-13, 16, 18, 24, 36-38, 43, 44, 47-49, 52, 53, 55, 59, 60, 63-65, 67, 68, 73, 74, 79 and 82 (write journal names properly); no. 15 (correct Burden of Disease); no. 80 (needs proper quoting) etc. Further many journal names are written full while other in abbreviations? Ref. no 25, 32, 80 and 83 are not complete add either their websites or other details. In ref no. 51 add country name after Sebelas Maret University. Authors are suggested to do changes in different font color in the revised R3 manuscript. | Thank you very much for reviewing our manuscript and providing interesting comments on how to improve it. We have now carefully edited the reference section according to the PLOS ONE style. The references have now been modified and updated as well. All authors gratefully acknowledge all of your comments | See the cleaned-up manuscript (R3). |
| 2. As suggested earlier, In Table 1 instead of using word “Sex” better to use “Gender”, what is authors opinion about the change? | We have now changed the word "sex" to "gender" in the table. | See the cleaned-up manuscript (R3). |
| 3. The overall sample size for this study was 634 (lines 144 and Table 1) but authors analyzed 625 questionnaires (lines 288) what was the reason for 98.6% response rate can be briefly added in Socio-demographic characteristics of study participants section. | Thank you for your helpful comments. We have now briefly explained the reason for the 98.6% response rate in the Socio-demographic characteristics of study participants section. | See the cleaned-up manuscript (R3). |
| 4. Author’s recommendations in lines 446-450 are OK but some sort of intervention or solution can be expected from Environmental and Occupational and Physiotherapy departments in the College of Medicine and Health Science of the University of Gondar, Ethiopia! Authors can suggest some basic exercises and correct sitting postures to the studied shopkeepers for a time period so that the purpose of “Community Service” for health professionals can be achieved. | Dear reviewers, thank you for raising this important issue. All the authors have accepted your comment and recommended what is expected of them accordingly. We hope that you are now interested in our revised manuscript. | See the cleaned-up manuscript (R3). |

**We gratefully acknowledge every one of your insightful and incredibly intriguing remarks.**

**We look forward to your positive response as soon as possible.**

With best regards,

Sincerely yours,

Authors
