# Supplementary material for: Prevalence and risk factors of work-related musculoskeletal disorders among shopkeepers in Ethiopia: Evidence from a workplace cross-sectional study
Source: PLoS One. 2024 Mar 21;19(3):e0300934. doi: 10.1371/journal.pone.0300934 (PMC10956884; doi:10.1371/journal.pone.0300934)
Supplement: Attachment — Submitted filename: Response to Reviewers.docx [file pone.0300934.s010.docx]

**Responses to the review’s comments**

**Dear PLOS ONE editorial team,**

Thank you for giving us the opportunity to submit a revised draft of the manuscript, and we would also like to thank you for your crucial comments on our paper (Manuscript ID: **PONE-D-22-30367R3**). We are very concerned and have combined all the suggested comments provided, which we believe strengthen our paper, and we hope this will render our paper eligible for consideration for publication in your reputed journal. We appreciate the time and effort that you and the reviewers dedicated to providing feedback on our manuscript and are grateful for the insightful comments and valuable improvements to our paper.

The authors would like to inform you that we have addressed the comments and recommendations made by **Reviewer #3** and the editor, point by point. In addition, throughout our revision, we made our best corrections too. All changes made to the original version are highlighted using tracking changes and attached as “**Revised Manuscript with Track Changes**." The unmarked copy of the manuscript is also attached as “**Manuscrip**t.” In addition, please see below a rebuttal letter that responds to each point raised by the academic editor and reviewers, and this letter is also attached to the submission as “**Response to Reviewer**."

**Response to editor’s comments**

1… Please review your reference list to ensure that it is complete and correct. If you have cited papers that have been retracted, please include the rationale for doing so in the manuscript text or remove these references and replace them with relevant current references. Any changes to the reference list should be mentioned in the rebuttal letter that accompanies your revised manuscript. If you need to cite a retracted article, indicate the article’s retracted status in the References list and include a citation and full reference for the retraction notice.

**Author response:** Dear the academic editor, Thank you for your comments regarding the reference list. We appreciate your attention to detail. We would like to clarify that we did not cite any retracted references in our manuscript.

If there is any confusion or specific reference that you believe to be retracted, please provide us with the details so that we can address the issue promptly and make any necessary corrections.

**Response to reviewers’ comments**

**Reviewer #3:** Dear Author,

1…… I am pleased to evaluate this manuscript entitled “Prevalence and risk factors of work-related musculoskeletal disorders among shopkeepers in Northwestern Ethiopia: evidence from a workplace cross-sectional study”.

The manuscript highlights an important area of concern and has a good methodology. However, it would be beneficial to present the information in a more concise and standard language. I recommend consulting the PLOS ONE manuscript preparation guide and other articles published in the journal for guidance on how to improve the presentation of your work.

**Author response:** Dear reviewer, we are very grateful for your general comments and suggestions that we believe it could improve the quality of this paper. Thank you for your positive feedback on the importance of the topic and the methodology used in our manuscript. We appreciate your suggestion to present the information in a more concise and standard language.

Based on your comments,

- We have now revised the manuscript according to the journal's guidelines and style.
- We have carefully reviewed the PLOS ONE Manuscript Preparation Guide, as well as other articles published in the journal, and have improved the presentation of our work accordingly.

2…. Based on the nature of the outcome variable, I recommend reanalyzing the data using the appropriate statistical analysis method. The comments and questions are forwarded in the comment boxes.

The Poisson regression is used when the outcome is a count variable. Please The logistic regression was reported on a previous version of the manuscript.

- What made you change to Poisson regression?
- Have you counted work-related musculoskeletal in the last 12 months and tried to identify the factors associated with each count (1, 2, 3 times in the last 12 months or week)?

**Author response:** Dear reviewer, thanks for your valuable comments and suggestions to merge these sentences, and we shared your comments and revised it accordingly. What made you change to Poisson regression? Dear reviewer, thank you for your comment. The reason we changed logistic regression to Poisson regression is to estimate the effect size of our outcome in PR rather than OR. In our previous early revision one (**PONE-D-22-30367**), one of the reviewer's comments was: "*Why logistic regression as the method and OR as the effect size? As the authors reported a prevalence of 81.1%, it would have been good to report the prevalence ratio instead of the OR. As it is, the OR overestimates the PR and the way the authors interpreted the OR is not correct*".

All of us authors were very convinced and accepted reviewer 1's comment and changed our analysis from logistic regression to Poisson regression to calculate the effect size in PR instead of OR. We appreciate your feedback and have decided to switch from logistic regression to Poisson regression for several reasons. Firstly, using Poisson regression allows us to estimate the effect size of our outcome in terms of the prevalence ratio (PR) rather than the odds ratio (OR). This change is in line with the suggestion of one of the previous reviewers who questioned why we used logistic regression and OR when we reported a prevalence rate of 81. 1%. They recommended reporting the effect size using PR instead of OR, as this overestimates the effect size. Taking this feedback into account, we have carefully revised our analysis and decided to use Poisson regression to calculate effect sizes in terms of PR. We believe that this change will improve the validity and interpretability of our results.

We have cited a scientific article in our main manuscript to support this (*Espelt A, Marí-Dell’Olmo M, Penelo E, Bosque-Prous M: Applied Prevalence Ratio estimation with different Regression models: An example from a cross-national study on substance use research. Adicciones 2016, 29(2):105-112*). Thank you again for your valuable input.

NB: The comments forwarded in the comment boxes have been appreciated and accepted. We have made the necessary corrections in our revised manuscript accordingly. All authors sincerely acknowledge and appreciate all your comments.

**Reviewer #4:** A good research work which can be repeated after implementing recommendations. Intervention in always an important part of research. All the best.

**Author response:** We are very grateful for your comments and time throughout the review process.
